# Supplementary material for: 3D designed and printed chemical generators for on demand reagent synthesis
Source: Nat Commun. 2019 Dec 2;10:5496. doi: 10.1038/s41467-019-13328-6 (PMC6889270; doi:10.1038/s41467-019-13328-6)
Supplement: Supplementary file 2 — Supplementary Information [file 41467_2019_13328_MOESM2_ESM.pdf]

Supplementary Information

3D-Designed and Printed Chemical Generators for On-Demand Reagent Synthesis

Sergey Zalesskiy,[1] Philip J. Kitson,[1] Przemyslaw Frei[1], Andrius Bubliauskas[1] and Leroy Cronin[1*]

*Correspondence to: lee.cronin@glasgow.ac.uk.

[1]*School of Chemistry, The University of Glasgow, Glasgow G12 8QQ (UK)*

Supplementary Methods 3
1. Traditional (Glassware) Synthesis of Target Materials 3
1.1 Synthesis of(NHS-diazirine) (succinimidyl 4,4'-azipentanoate) 3
1.2 Synthesis of Tris(dibenzylideneacetone)dipalladium(0)-chloroform adduct 10
1.3 Synthsis of 1,1,1-Triacetoxy-1,1-dihydro-1,2-benziodoxol-3(1H)-one (Dess Martin Periodinane) 15
1.4 Synthesis of{P_8W_{48}} 21
2. Reactionware Cartridge Design and Synthesis of Target Materials 24
2.1 General Remarks 24
2.2 Synthesis of (NHS-diazirine) (succinimidyl 4,4'-azipentanoate) 24
2.3 Synthesis of tris(dibenzylideneacetone)dipalladium(0) 40
2.4 Synthesis of 1,1,1-triacetoxy-1,1-dihydro-1,2-benziodoxol-3(1H)-one (Dess Martin Periodinane) 50
2.5 Synthesis of {P_8W_{48}} 59
3. Product Validation Experiments 68
3.1 DMP validation. Oxidation of menthol to menthone.[6] 68
3.2 Pd_2dba_3 validation. Suzuki coupling. 68
3.3 NHS-Diazirine reactivity validation. 69
3.4 {P_8W_{48}} validation – formation of a wheel-shaped Cu_{20} tungstophosphate 69
Supplementary References 71

Supplementary Methods

Solvents and reagents were used as received from commercial suppliers unless otherwise stated. Polypropylene feedstock for 3D printing was purchased from Barnes Plastic Welding Equipment Ltd., Blackburn, UK. 3D printing was achieved on Ultimaker 2+ FDM 3D printers supplied by Ultimaker and modified by the authors to print with polypropylene. ^{1}H, ^{13}C NMR spectra were recorded on a Bruker Avance III HD 600 MHz and Bruker Avance II 400 MHz spectrometers. Chemical shifts are reported in ppm relative to residual solvent (multiplicities are given as s: singlet, d: doublet, t: triplet, q: quartet, m: multiplet, with coupling constants reported in Hz). Mass Spectra were recorded on a Q-trap, time-of-flight MS (MicroTOF-Q MS) instrument equipped with an electrospray (ESI) source supplied by Bruker Daltonics Ltd. All analysis was collected in positive ion mode. The spectrometer was calibrated with the standard tune-mix to give a precision of ca.1.5 ppm in the region of m/z 100-3000. Percentage purity was assessed on a Dionex 3000 Ulitmate HPLC system comprising LPG-3400SD pump, DAD-3000 detector with a 13 µL flow cell, WPS-3000TFC analytical autosampler with fraction collector, and TCC-3000SD column thermostat, running Chromeleon 6.8. A reversed-phase C18 column (Purospher® STAR RP-18 endcapped (5 µm), 100 × 4.6 mm) was used.

1. Traditional (Glassware) Synthesis of Target Materials

1.1 Synthesis of(NHS-diazirine) (succinimidyl 4,4'-azipentanoate)

The synthetic procedure for the glassware synthesis of (NHS-Diazirine) (succinimidyl 4,4'-azipentanoate) was adapted from a literature procedure.[1]

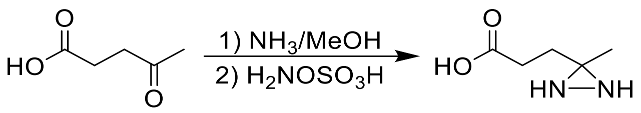

NOTE: Take precautions to avoid exposure to light throughout the synthesis. Levulinic acid (0.5 g, 4.31 mmol) was dissolved in anhydrous MeOH (2 mL) and transferred into 100 mL round bottom flask. The reaction vessel was placed in a cooling bath at -15°C. A 7N solution of ammonia in methanol (7.4 ml, 52 mmol, 12 equiv) was added into the reaction vessel, followed by 3Å molecular sieves (3 g). The mixture was stirred under nitrogen atmosphere for 3 h. Hydroxylamine-O-sulfonic acid (0.535 g, 4.73 mmol, 1.1 equiv) was dissolved in 4 mL of anhydrous MeOH and added into the reaction vessel dropwise while maintaining the temperature of the reaction at -15°C. The reaction was then stirred for 16 h, while its temperature was allowed to increase to ambient level. Subsequently the suspension was filtered off and the resultant filtrate concentrated *in vacuo* (at 20°C and 50 mbar), to afford oily off-yellow residue of the crude product which was used in the next step without purification.

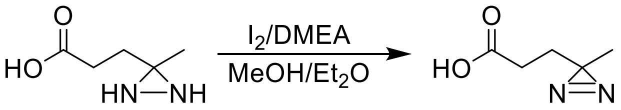

Crude diaziridine was dissolved in anhydrous MeOH (2 mL) and stirred on an ice bath for 5 min. N,N-dimethylethylamine (0.315 g, 4.30 mmol, 1.03 equiv) was added into the solution and this was stirred for further 5 min. Beads of iodine were then added gradually into the reaction mixture. When adding the iodine, the colour of the reaction mixture became brown initially, but within a few seconds the colour disappeared to give a colourless solution again. Upon further addition of iodine, the rate of the disappearance of the brown colour slowed significantly. When the brown colour began to fade within 7 min of the last addition, giving yellow tinted solution at the end of the 7 min period, the addition of iodine was stopped. Finally, the reaction was left to stir for another 30 min without further addition of iodine. 2 mL of 50 % w/v of KI solution was then added into the reaction, followed by 0.1 mL of saturated solution of ascorbic acid and 0.3 mL of 3 M HCl added dropwise. The mixture was stirred for 5 min and then extracted twice with 20 mL diethyl ether. The organic phase was dried over magnesium sulfate and the solvent evaporated in vacuo to afford the product as a clear yellow oil, mass (0.248 g, 1.93 mmol), yield 45%, purity 89%. ^{1}H NMR (600.1 MHz; 303 K; $CDCl_3$; δ, ppm; *J*, Hz): 10.89 (1H, bs), 2.22 (2H; t; 7.6), 1.70 (2H; t;7.6 Hz), 1.03 (3H; s); ^{13}C{^{1}H} NMR (150.9 MHz, 303 K, $CDCl_3$; δ, ppm): 178.9, 29.5, 28.7, 25.2, 19.8.

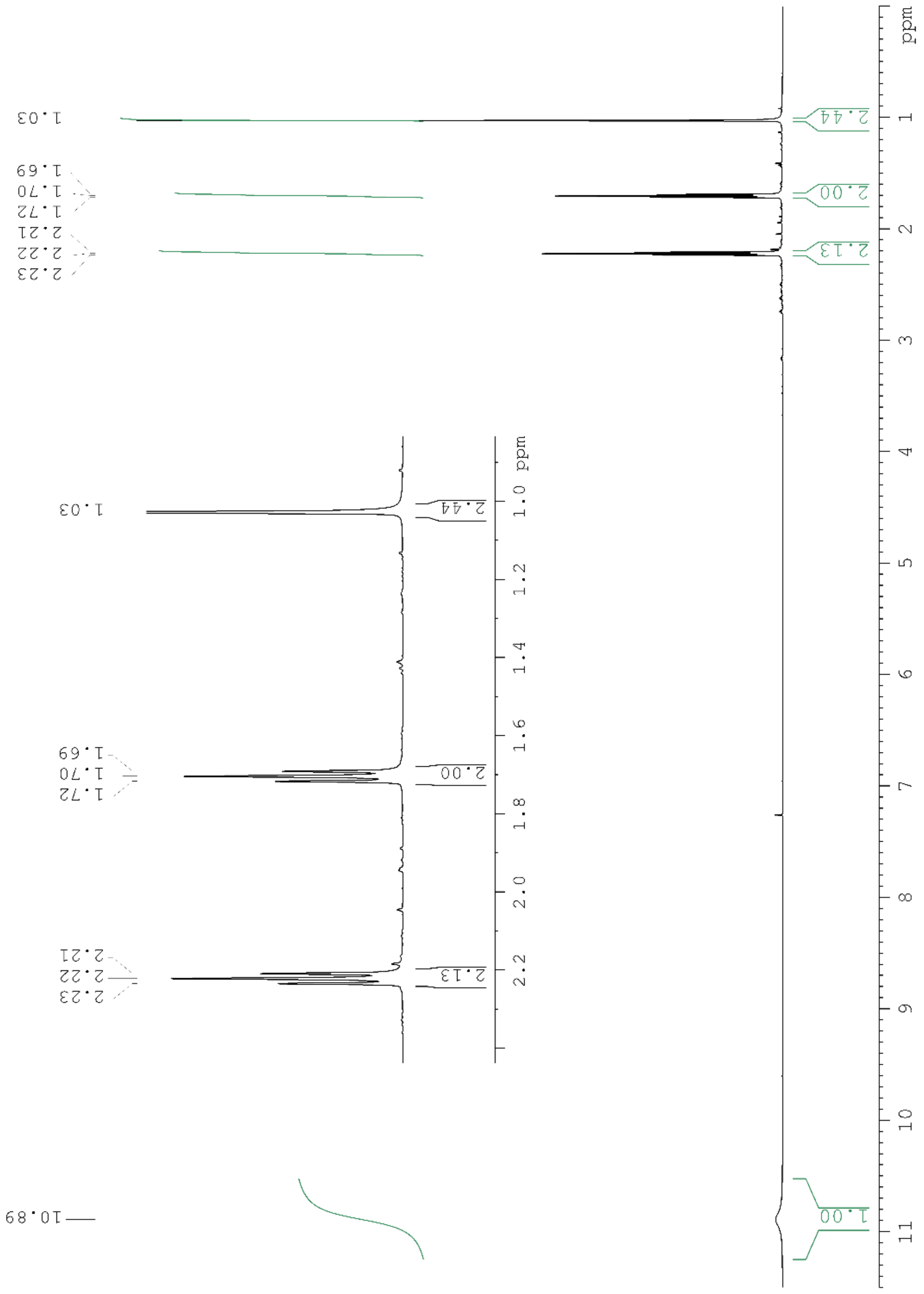

Supplementary Figure 1: ^{1}H NMR of 3-(3-methyl-3H-diazirin-3-yl)propanoic acid.

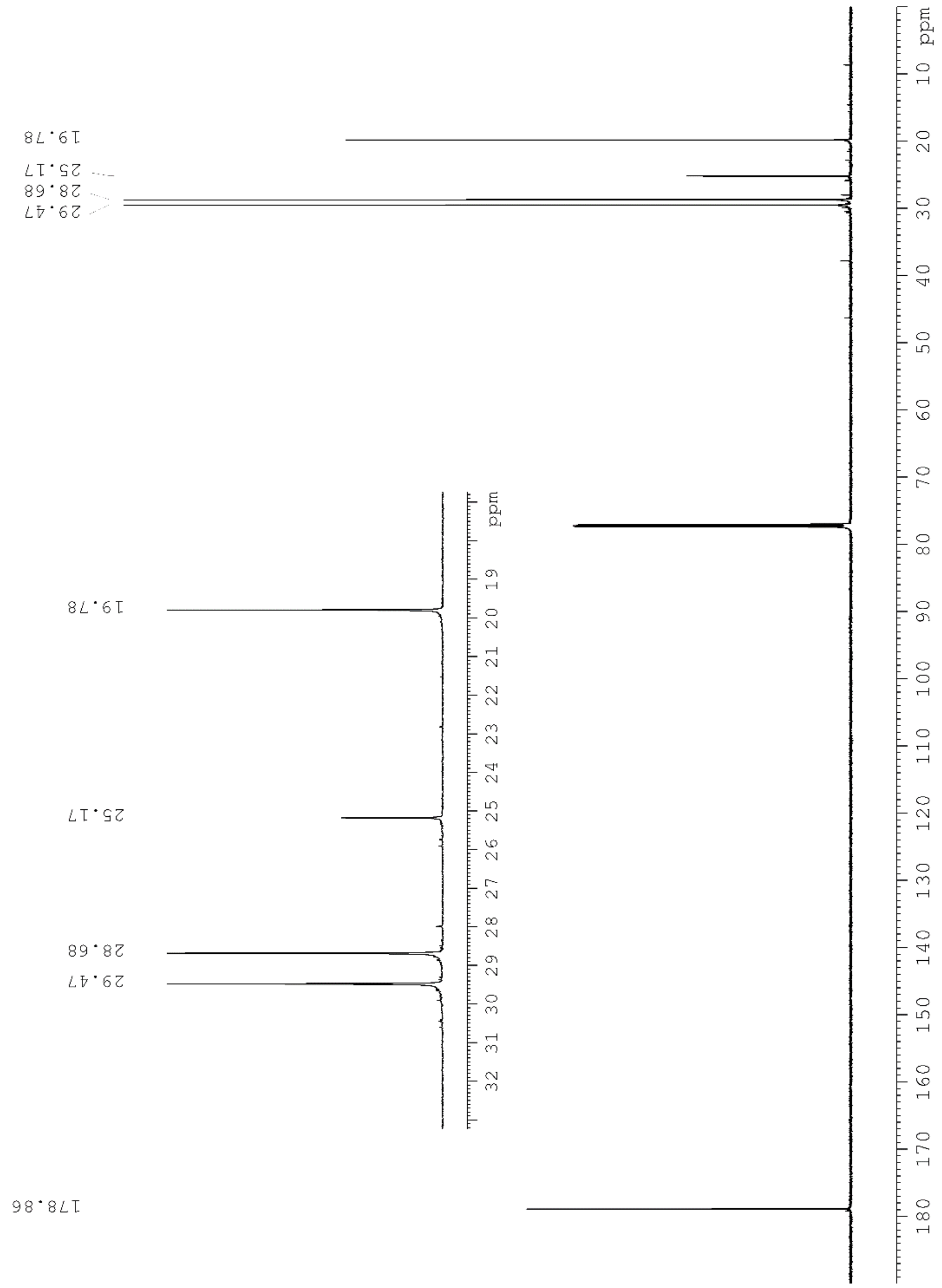

Supplementary Figure 2: $^{13}C\{^{1}H\}$ NMR of 3-(3-methyl-3H-diazirin-3-yl)propanoic acid.

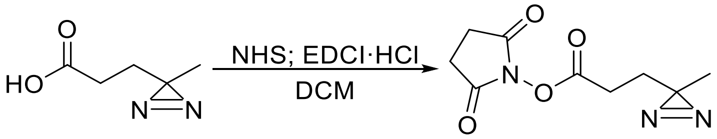

The starting material (0.248 g, 1.93 mmol) was dissolved in anhydrous DCM (4 mL) and stirred on an ice bath. *N*-(3-dimethylaminopropyl)-*N*′-ethylcarbodiimide hydrochloride (0.390 g, 2.03 mmol, 1.05 equiv) was then added into the reaction vessel along with *N*-hydroxysuccinimide (0.223 g, 1.93 mmol, 1 equiv). The reaction mixture was then stirred for 20 h under nitrogen atmosphere. The temperature of the reaction was allowed to return to ambient level during this time, while exposure to light was limited at all times. After the completion, the crude product was washed twice with 2 mL of water. The organic phase was dried over magnesium sulfate and the solvent evaporated in vacuo to afford final product as yellow solid. The crude was re-dissolved in a mixture of 2.5 mL anhydrous diethyl ether and 0.5 mL of anhydrous methanol. This was left overnight at 2°C. The following day the mother liquor was withdrawn from the vessel, leaving behind colourless crystals which upon drying under reduced pressure become off-white. The mass of the product was (0.313 g, 1.39 mmol), yield 72%, purity 94%. ^{1}H NMR (600.1 MHz; 303 K; $CDCl_3$; δ, ppm; *J*, Hz): 2.83 (4H, s), 2.51 (2H; t; 7.8), 1.80 (2H; t; 7.8), 1.07 (3H; s); $^{13}C\{^{1}H\}$ NMR (150.9 MHz, 303 K, $CDCl_3$; δ, ppm): 169.1, 167.7, 29.7, 25.9, 25.7, 24.9, 19.6.

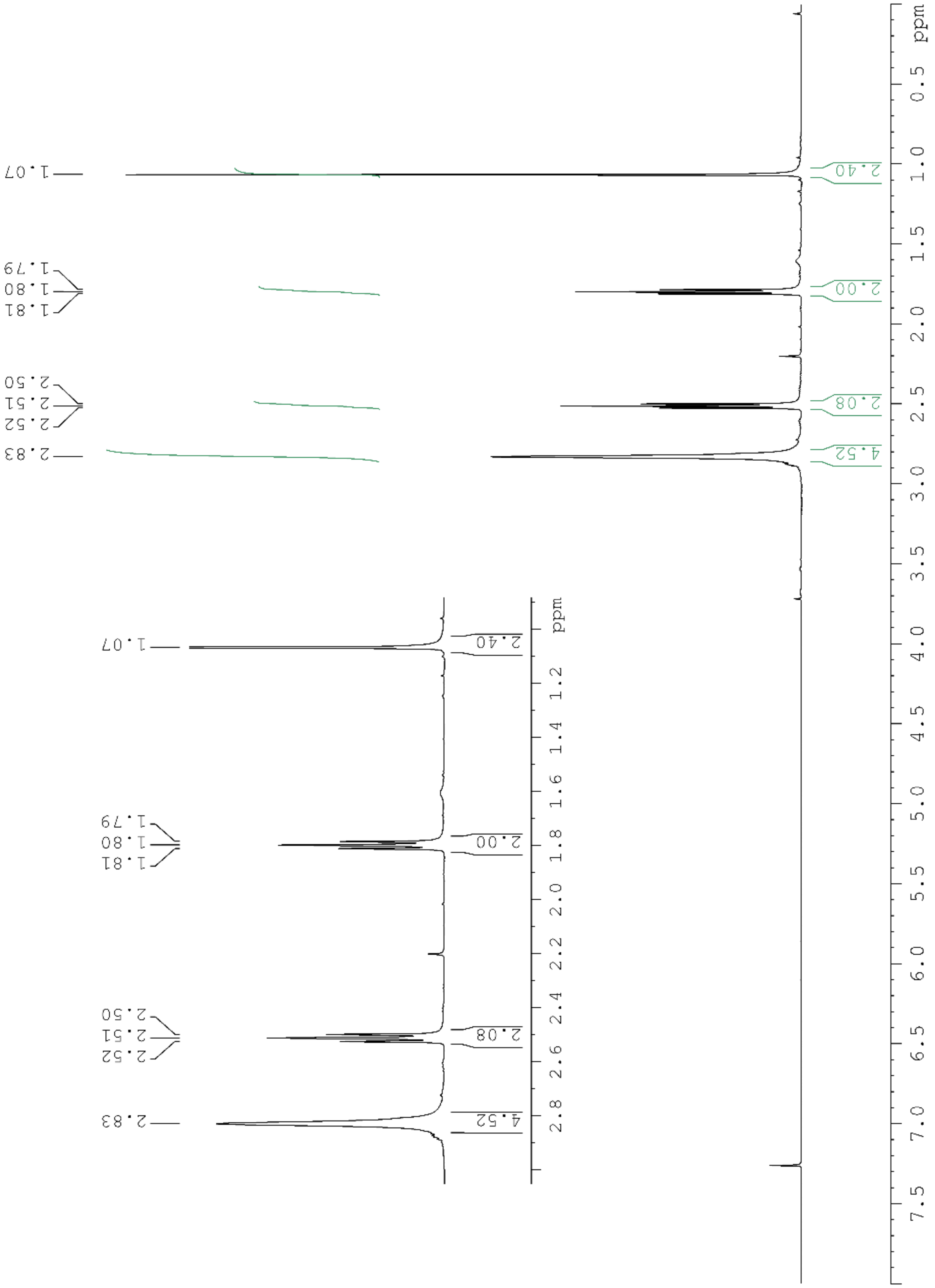

Supplementary Figure 3: ^{1}H NMR of (NHS-diazirine) (succinimidyl 4,4'-azipentanoate).

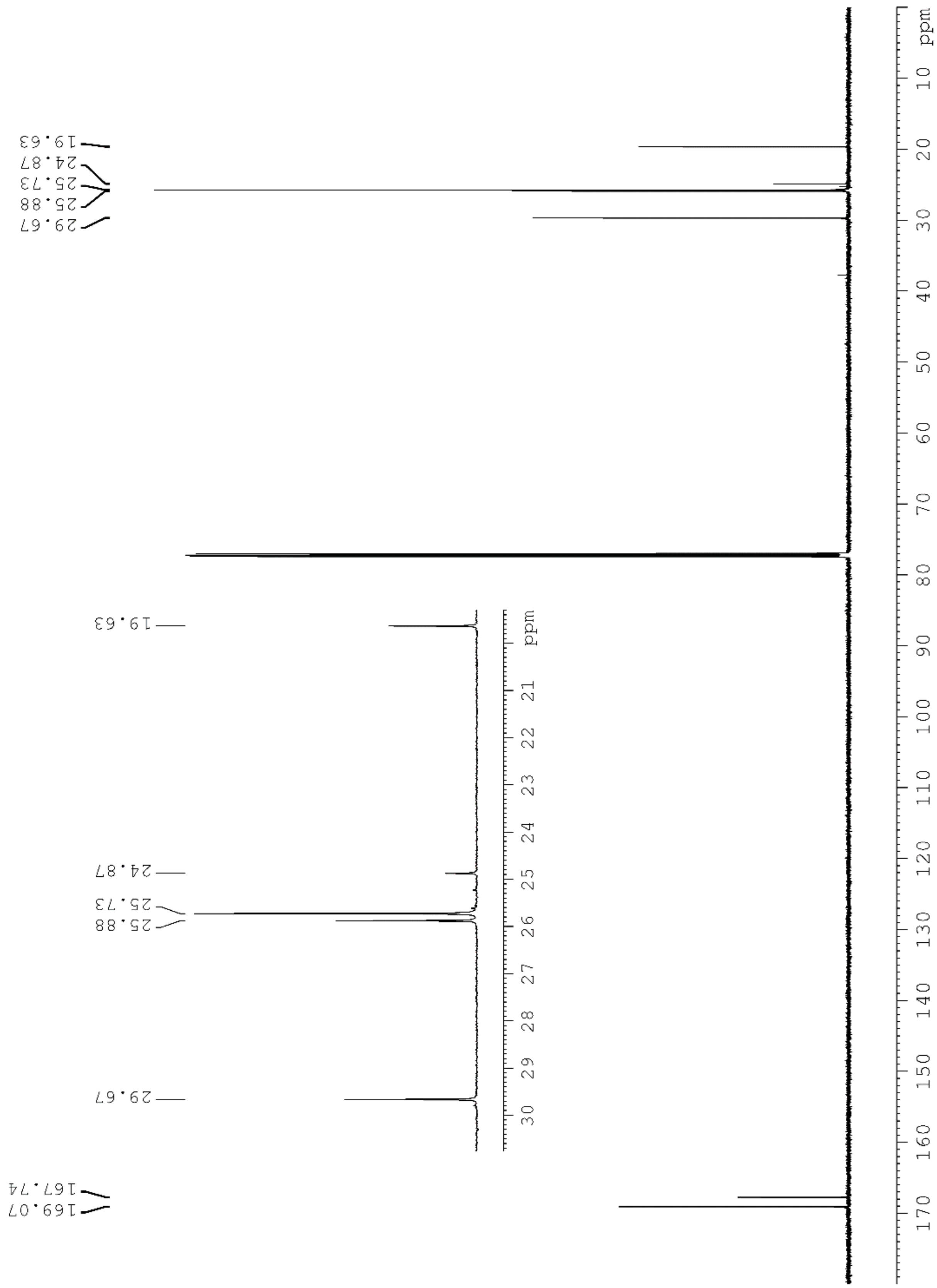

Supplementary Figure 4:$^{13}C\{^{1}H\}$ NMR of (NHS-diazirine) (succinimidyl 4,4′-azipentanoate).

1.2 Synthesis of Tris(dibenzylideneacetone)dipalladium(0)-chloroform adduct

The synthetic procedure for the glassware synthesis of tris(dibenzylideneacetone)dipalladium(0)-chloroform adduct was adapted from literature procedures.2,3 To prevent decomposition of the Pd2dba3, chloroform used for the synthesis was purified to remove the possible traces of HCl. This was achieved by washing of the chloroform three times with deionised water, followed by pre-drying with magnesium sulfate and finally refluxing over phosphorus pentoxide for one hour, followed by distillation. The freshly distilled chloroform was stored over well dried 3Å molecular sieves, in a dark space.

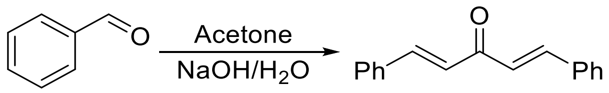

Benzaldehyde (1.0 g, 9.4 mmol, 2 equiv) was loaded into a round bottom flask, followed by HPLC grade acetone (0.27 g, 4.7 mmol, 1 equiv) and 6 mL of EtOH. This mixture was stirred, while 10% w/v NaOH (8.48 mL, 21.2 mmol, 4.5 equiv) was added dropwise over the course of 30 min. The reaction was stirred for further 2 h. The yellow precipitate of the product was then filtered off and washed with 12 mL of water. The precipitate was dried thoroughly under vacuum to give the crude which was then re-dissolved in ethyl acetate and recrystallized to afford pure dibenzylideneacetone. The mass of the product was (0.982 g, 4.19 mmol), yield 89%, purity 91%. ^{1}H NMR (600.1 MHz; 303 K; $CDCl_3$; δ, ppm; *J*, Hz): 7.75 (2H; d; 15.9), 7.64 – 7.61 (4H; m), 7.44 – 7.41 (6H; m), 7.09 (2H; d; 15.9); $^{13}C\{^{1}H\}$ NMR (150.9 MHz, 303 K, $CDCl_3$; δ, ppm): 189.1, 143.5, 135.0, 130.7, 129.1, 128.6, 125.6.

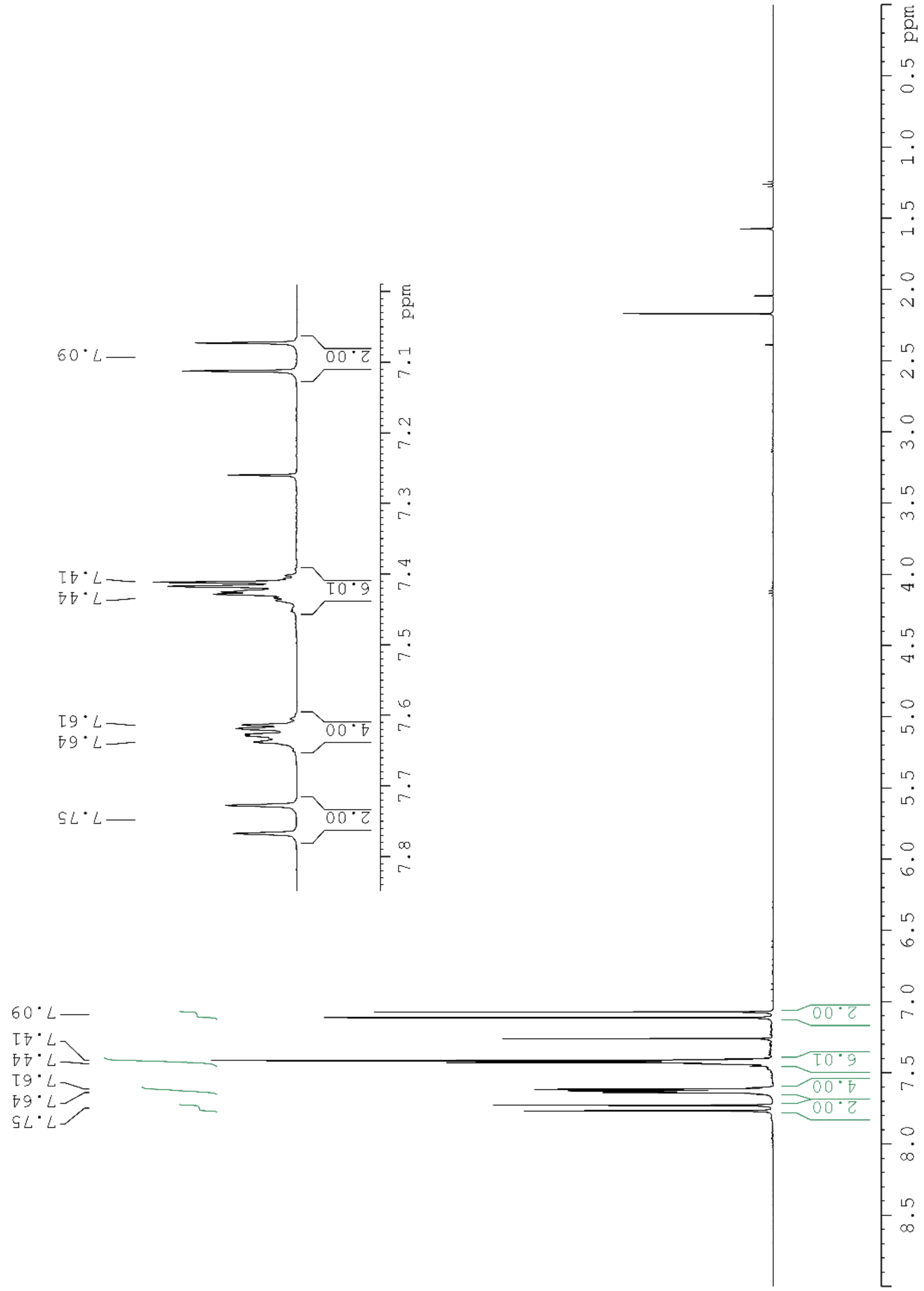

Supplementary Figure 5: ^{1}H NMR of dibenzylideneacetone.

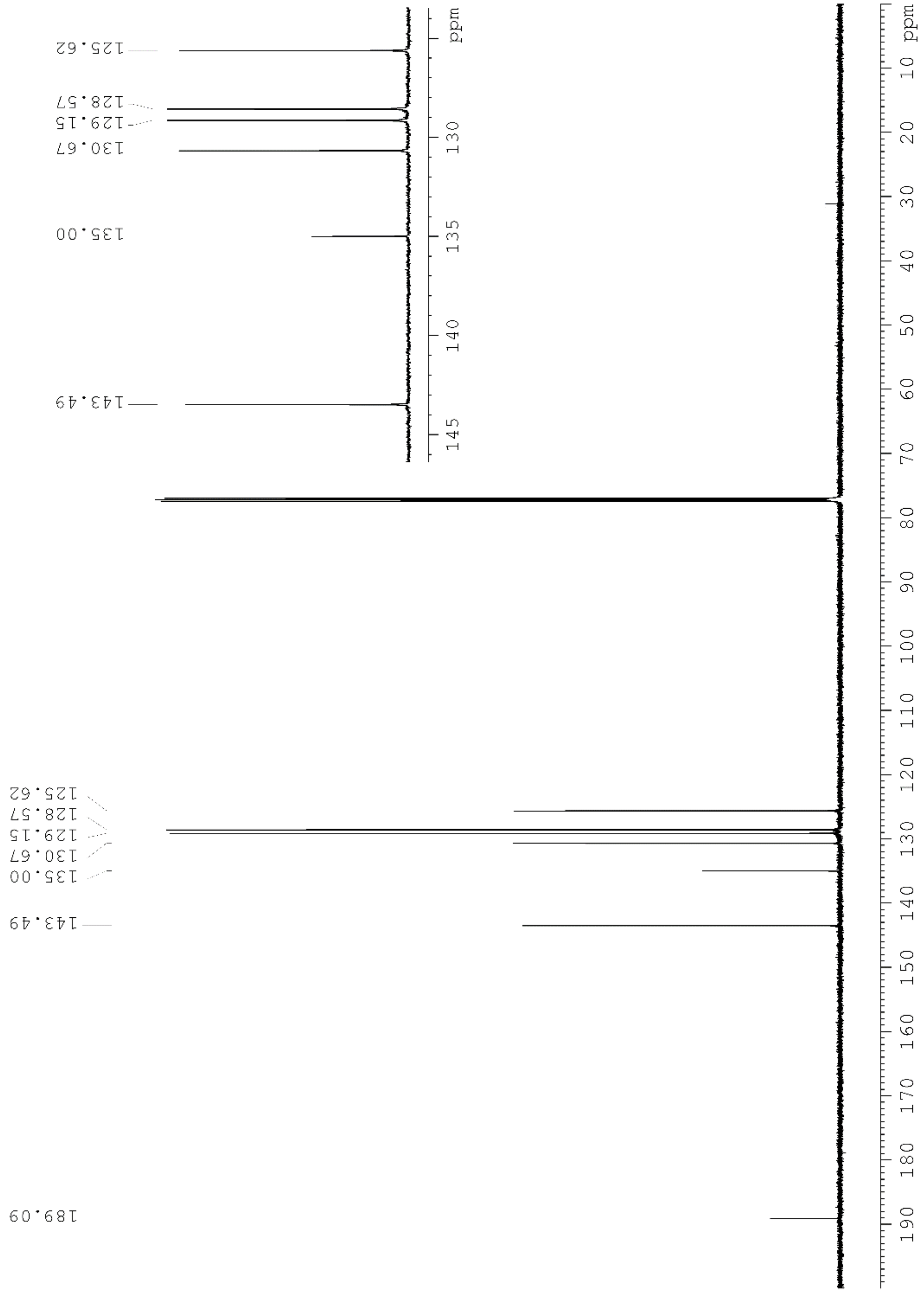

Supplementary Figure 6: $^{13}C\{^{1}H\}$ NMR of dibenzylideneacetone.

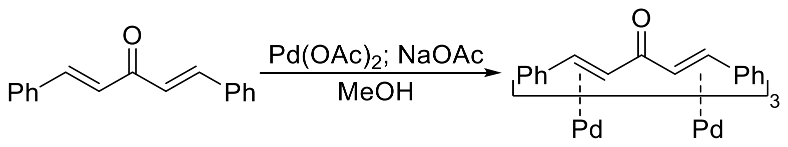

Palladium acetate (101 mg , 0.451 mmol) was loaded into a round bottom flask, followed by sodium acetate (370 mg, 4.511 mmol, 10 equiv), dibenzylideneacetone (211.3 mg, 0.902 mmol, 2 equiv) and 10 mL of anhydrous methanol. This mixture was stirred for 3 h under nitrogen atmosphere in a heat stabilised water bath at 40°C. During this time dark brown precipitate was formed. After cooling to room temperature, the precipitate was filtered off and washed three times with 3 mL of water and twice with 3 mL of methanol. The crude was dried under vacuum and then re-dissolved in 25 mL of dry chloroform. Note that acid traces were removed from the chloroform before use. The solvent was removed in vacuo at 40°C and the solid residue was re-dissolved in 5 mL of dry chloroform and to this solution was added 20 mL of HPLC grade acetone. The resultant mixture was kept at -18°C overnight. The next day the dark brown solid was filtered off and washed twice with 5 mL of cold acetone. The product was dried under vacuum, giving mass of (0.190 g; 1.84 mmol), 84% yield, 90% purity.[3] ^{1}H NMR (600.1 MHz; 303 K; $CDCl_3$; δ, ppm): Major isomer – 6.80, 6.73, 6.64, 6.44, 6.37, 6.15, 5.93, 5.88, 5.33, 4.98, 4.93; Minor isomer – 6.03, 5.65, 5.03, 4.90, 4.85.

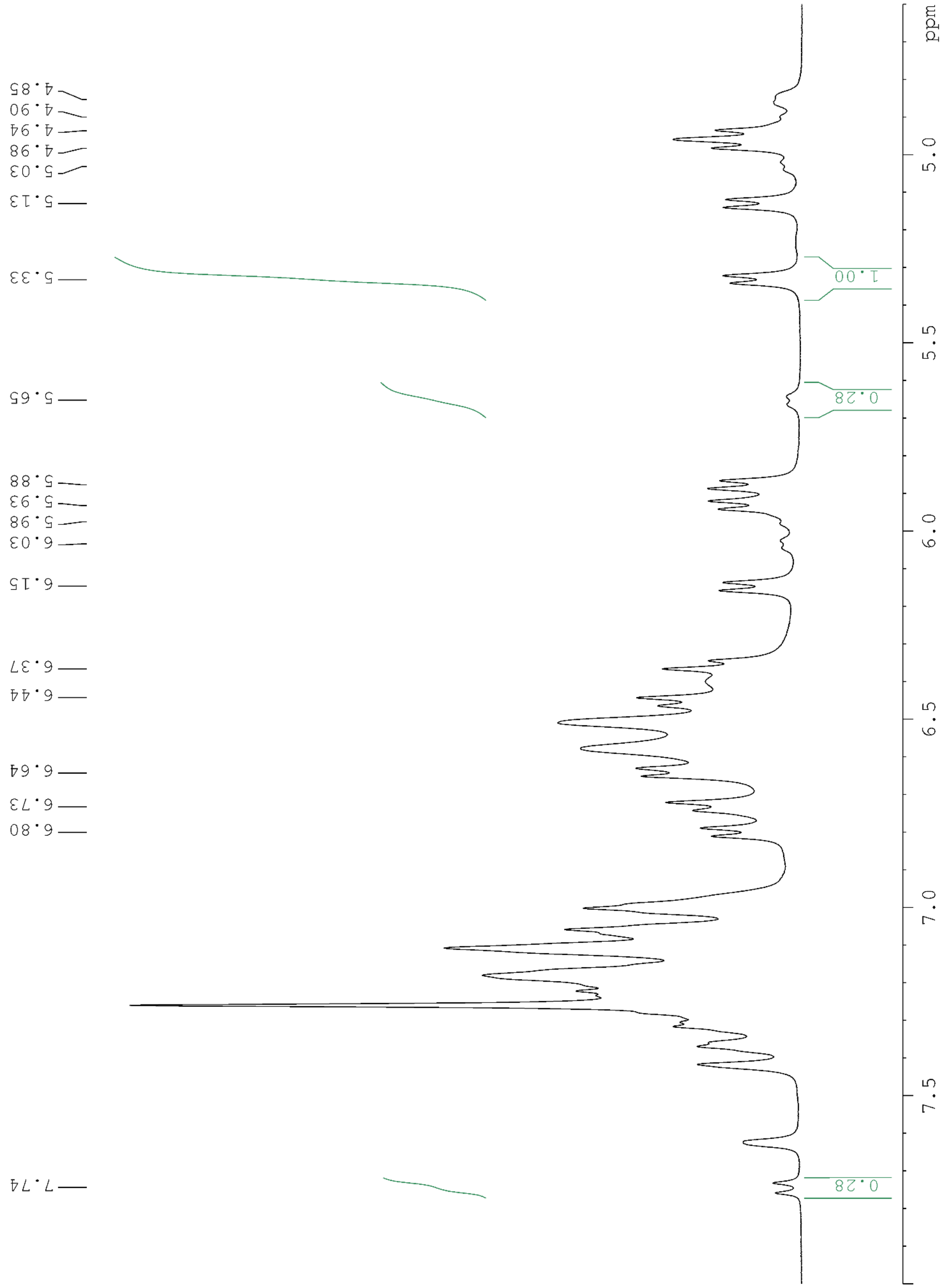

Supplementary Figure 7: ^{1}H NMR of tris(dibenzylideneacetone)dipalladium(0)-chloroform adduct.

1.3 Synthsis of 1,1,1-Triacetoxy-1,1-dihydro-1,2-benziodoxol-3(1H)-one (Dess Martin Periodinane)

The synthetic procedure for the glassware synthesis of Dess-Martin Periodinane was adapted from the literature[4] and adapted accordingly to suit the needs of this project.

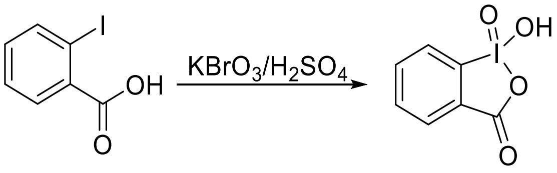

2-iodobenzoic acid (2 g, 8.06 mmol) was loaded into a two neck round bottom flask equipped with a thermometer. 15 mL of 0.8M sulfuric acid was then added into the vessel. The resulting suspension was stirred vigorously and brought to 55°C. Potassium bromate was then added in portions (1.785 g, 10.69 mmol, 1.3 equiv) over a period of 40 min. The suspension was brought to 68°C and the reaction stirred for 3 h 40 min. Note that after the addition of potassium bromate any visible aggregates of KBrO3 were broken down with a glass rod. Any material which deposited on the glass rod and the walls of the round bottom flask was washed down into the reaction mixture with more sulfuric acid. 26 mL of sulfuric acid was used in total. Upon completion, the reaction vessel was cooled to room temperature. Filtration and subsequent washing with 30 mL of water, followed by 12 mL of ethanol and finally 12 mL of anhydrous diethyl ether. The product in the form of a white powder was then dried in a desiccator, overnight. The mass of the product was (2.045 g, 7.30 mmol), yield 91%, purity estimated by ^{1}H NMR >90%. This material was used in in the next step without further purification. ^{1}H NMR (600.1 MHz; 303 K; $CDCl_3$; δ, ppm; *J*, Hz): 8.15 (1H; d; 7.9), 8.04 – 7.99 (2H; m), 7.84 (1H; t; 7.4); ^{13}C{^{1}H} NMR (150.9 MHz, 303 K, $CDCl_3$; δ, ppm): 167.4, 146.5, 133.4, 132.9, 131.4, 130.1, 125.0.

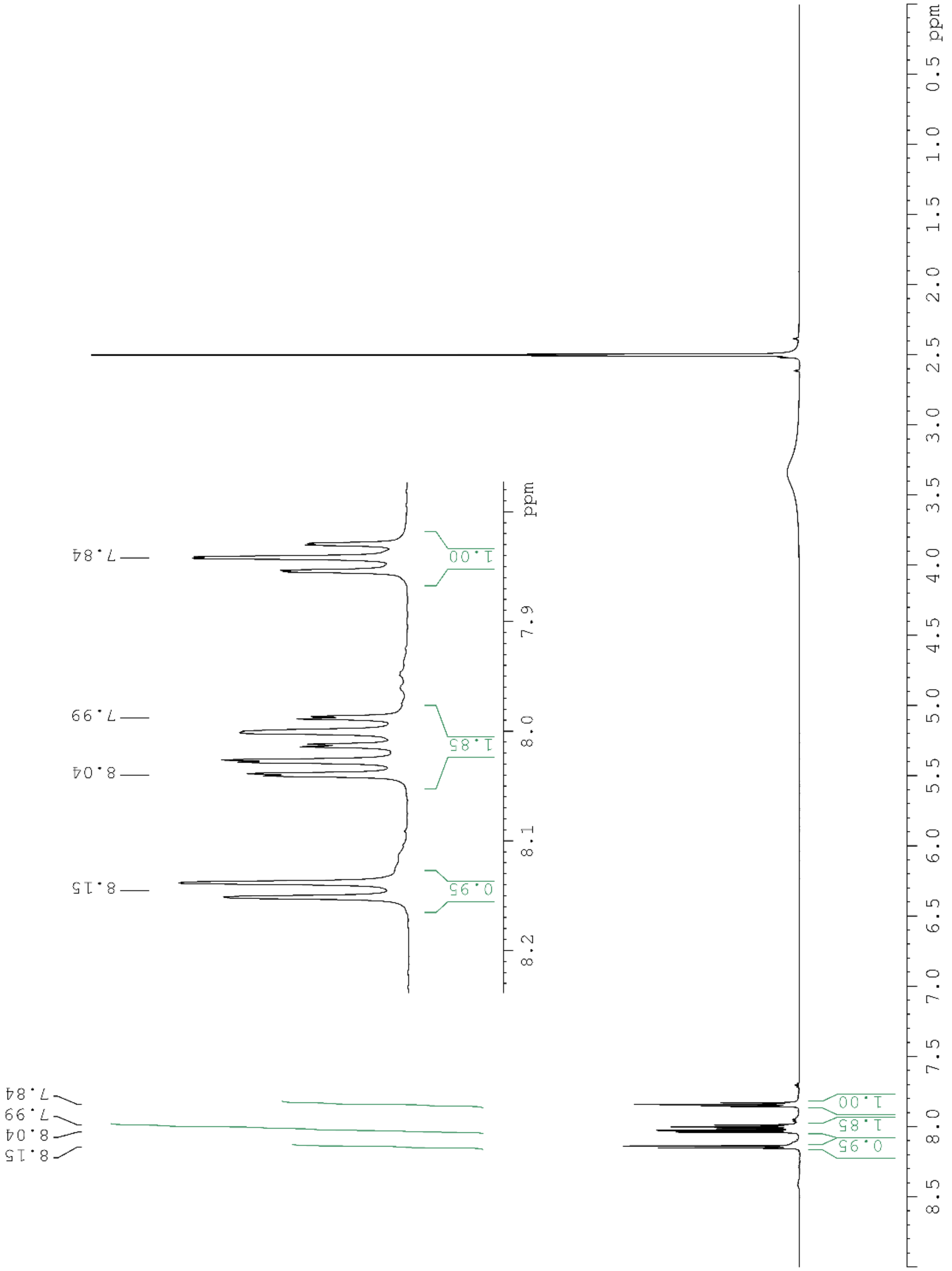

Supplementary Figure 8: ^{1}H NMR of 2-iodoxybenzoic acid.

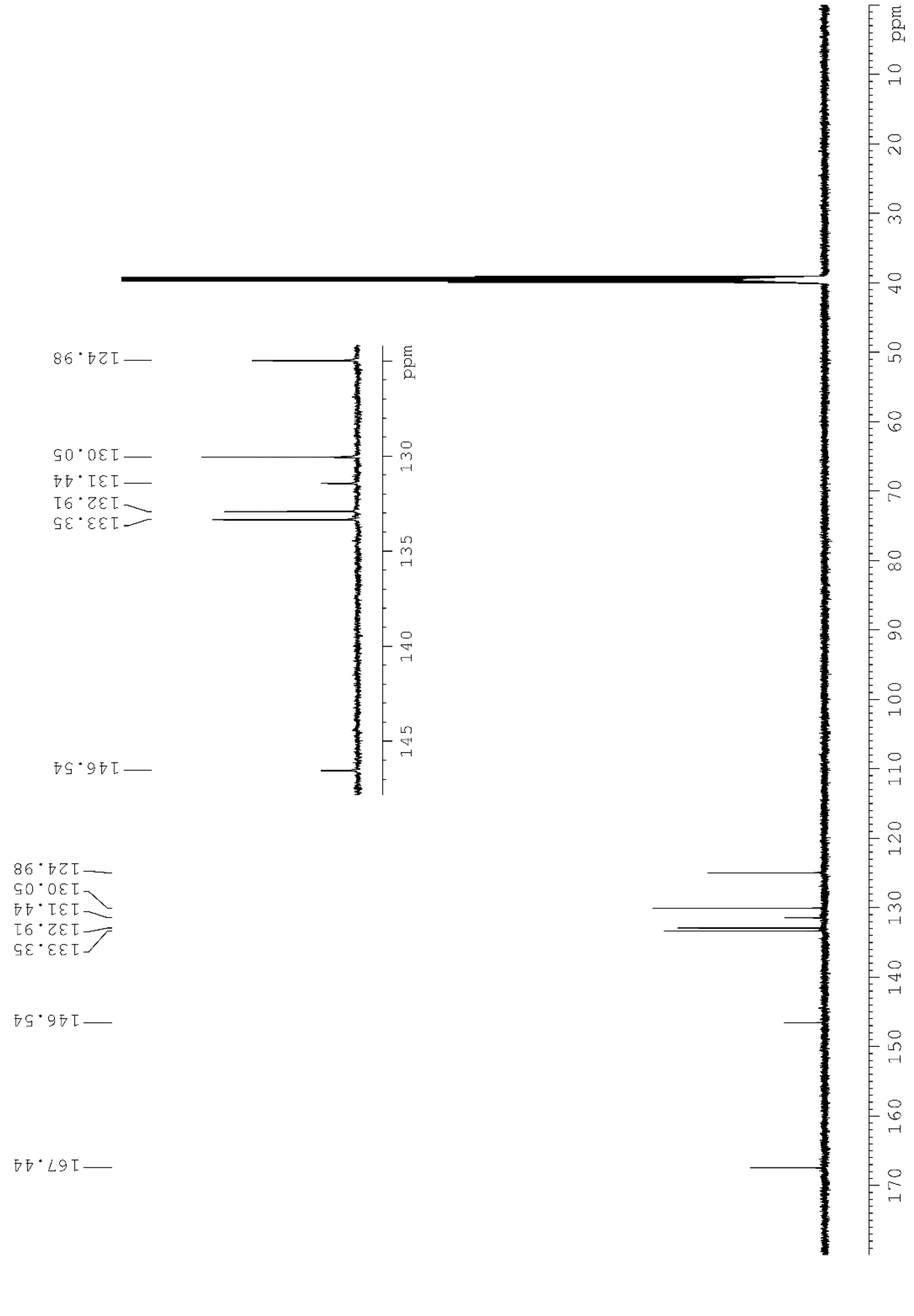

Supplementary Figure 9: $^{13}C\{^{1}H\}$ NMR of 2-iodoxybenzoic acid.

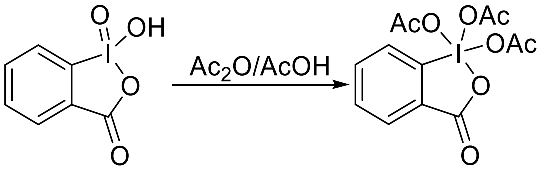

2-Iodoxybenzoic acid (2.045 g, 7.30 mmol) was transferred into a two neck round bottom flask equipped with a thermometer and a drying tube filled with fused calcium chloride. Acetic anhydride (4.869 g, 4.5 mL, 47.69 mmol, 6.5 equiv) was then added into the reaction vessel, followed by acetic acid (3.99 g, 3.8 mL, 66.44 mmol, 9.1 equiv). The mixture was heated up to 80℃ and stirred for 2 h. The stirring was then stopped, the reaction was placed under inert atmosphere and cooled at 2℃, overnight. The following day the product was filtered off under a flow of nitrogen gas. The resultant white crystalline material was washed with 12 mL of cold anhydrous diethyl ether and then dried thoroughly under vacuum. Mass 2.59 g, 6.126 mmol; yield 76%; purity 84%. ^{1}H NMR (600.1 MHz; 303 K; $CDCl_3$; δ, ppm; *J*, Hz): 8.25 (1H; d; 7.6), 8.22 (1H; d; 8.2), 8.00 (1H; t; 7.8), 7.83 (1H; t; 7.3), 2.26 (3H, s), 1.94 (6H, s); ^{13}C{^{1}H} NMR (150.9 MHz, 303 K, $CDCl_3$; δ, ppm): 175.9, 174.1, 166.2, 142.5, 135.8, 133.9, 132.0, 126.6, 126.2, 20.6, 20.4.

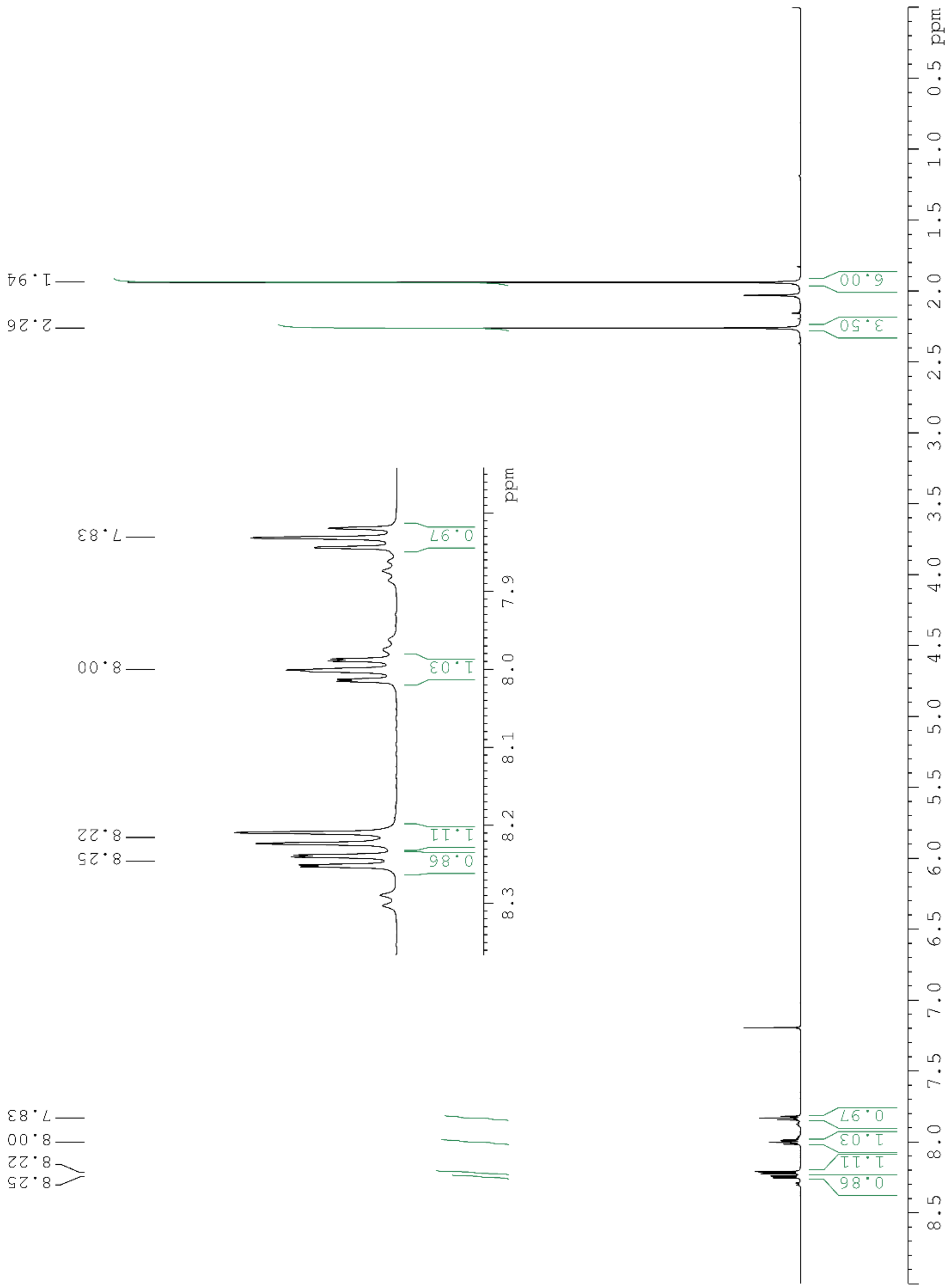

Supplementary Figure 10: ^{1}H NMR of 1,1,1-triacetoxy-1,1-dihydro-1,2-benziodoxol-3(1H)-one.

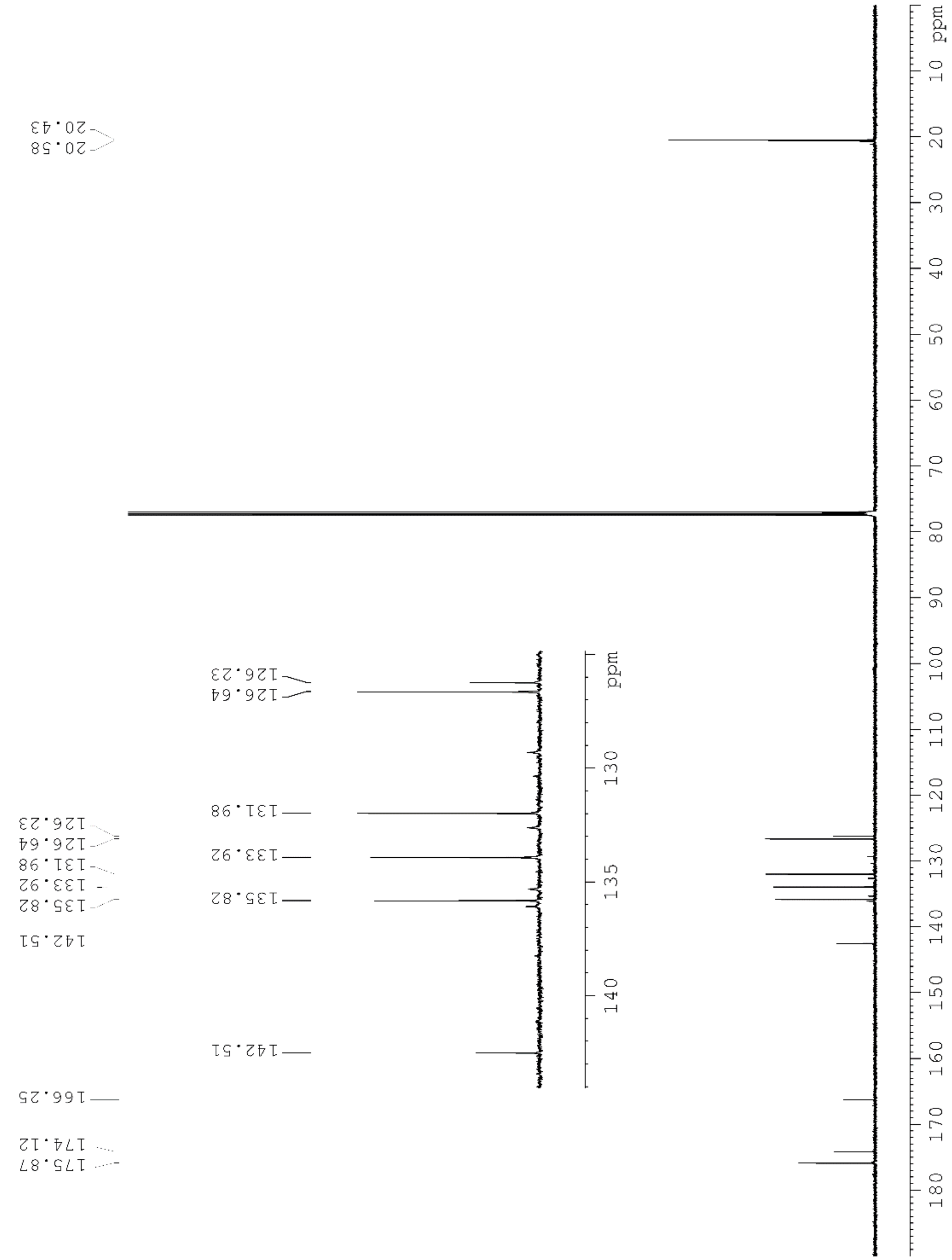

Supplementary Figure 11: $^{13}C\{^{1}H\}$ NMR of 1,1,1-triacetoxy-1,1-dihydro-1,2-benziodoxol-3(1H)-one.

1.4 Synthesis of{P_8W_{48}}

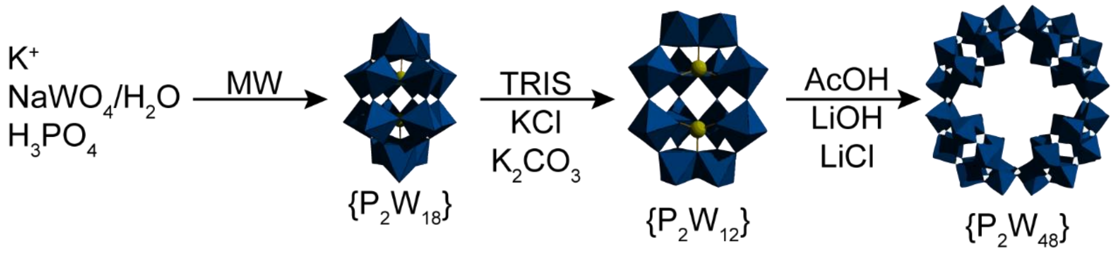

Supplementary Figure 12: Reaction scheme for the synthesis of {P_8W_{48}}.

{P_2W_{18}} was prepared based on a method developed in-house within the Cronin group by T. Boyd which was based on a procedure reported in the literature.[5] $Na_2WO_4 \cdot 2H_2O$ (300 g, 0.91 mol) was dissolved in 350 mL H_2O in a 1 L flask. Once completely dissolved, 250 mL of 4 M HCl was added at a rate of 2 drops per second from a 500 mL dropping funnel. After the addition of hydrochloric acid the pH of the clear solution was 7.21. Next, 250 mL 4M H_3PO_4 was added at a rate of 4 drops per second from the same funnel. The resulting clear, pale green solution (pH 0.89) was heated to 100 °C under reflux for 24 hours before transferring to a 1.5 L beaker. Once cooled, KCl (150 g, 2.01 mol) was added, and the reaction mixture was stirred for 10 minutes. A chalky green precipitate formed under stirring which was collected by Büchner filtration. After drying in air overnight, this powdery solid was transferred to a 1 L beaker and dissolved in 650 mL H_2O. The resulting clear green solution was heated to 80 °C in a 1 L round-bottomed flask for 3 days. A condenser was fitted to this flask for duration of the reflux. After the 3-day reflux period, the clear, green solution was transferred to a 1 L beaker and allowed to cool slowly back to room temperature on the bench. The solution was then placed in a temperature-controlled room (18 °C) for crystallization. Large, green block crystals, which began to form after a few hours, were collected by Büchner filtration after 3 days and dried overnight in a desiccator. The resulting crystals were re-dissolved in a minimum amount of boiling water and put in an ice bath to recrystallize. Yield: 139.2 g, 28.7 mmol, 57% based on W. ^{31}P NMR (242.9 MHz; 303 K; D_2O; δ, ppm): -13.0, -12.3, -11.5, -10.1. Element % weight calculated for $K_6[P_2W_{18}O_{62}]\cdot 14H_2O$: P, 1.28; W, 68.22; K, 4.84; Found: P, 1.22; W, 62.04; K, 4.48.

Supplementary Table 1. {P_2W_{18}} elemental ratio based on molar ratio from ICP data.

Sample	K	P	W
$K_6[P_2W_{18}O_{62}]\cdot 14H_2O$	6.117	2.107	18.000

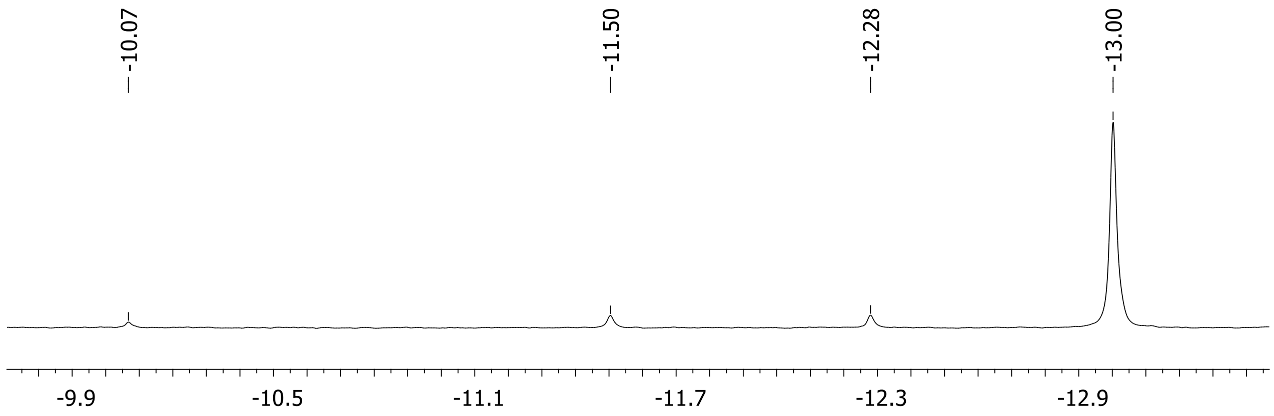

Supplementary Figure 13: ^{31}P NMR of $K_6[P_2W_{18}O_{62}]\cdot 14H_2O$ synthesised in glassware.

In a 1 L beaker a sample of 83 g (17 mmol) of $\{P_2W_{18}\}$ was dissolved in 300 mL H2O. A solution of 48.4g (400 mmol) of tris(hydroxymethyl)aminomethane in 200 mL of water was added. The solution was left at RT for 30 min and afterwards, 80 g of KCl was added. After complete dissolution, a solution of 55.3 g (400 mmol) of K_2CO_3 in 200 mL of water was added. The solution was vigorously stirred for 15 min followed by appearance of the white precipitate after a few minutes., The precipitate was vacuum-filtered using an S4 porosity sinter funnel and dried on the funnel for 18 hours. After that the precipitate was washed with 50 mL of ethanol and dried for another 3h. Yield: 58.4 g, 14.8 mmol, 85% based on W. ^{31}P NMR (242.9 MHz; 303 K; D_2O; δ, ppm): 2.48, -9.23. Element % weight calculated for $K_{12}[H_2P_2W_{12}O_{48}]\cdot 24H_2O$: P, 1.57; W, 56.01; K, 11.91; Found: P, 1.67; W, 55.42; K, 13.07.

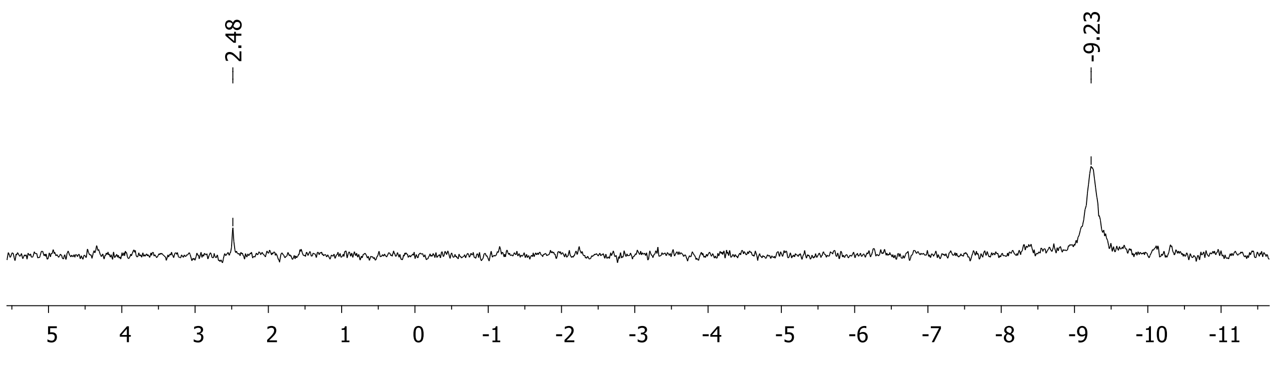

Supplementary Figure 14: ^{31}P NMR of $K_{12}[H_2P_2W_{12}O_{48}]\cdot 24H_2O$ synthesised in glassware.

Supplementary Table 2: $\{P_2W_{12}\}$ elemental ratio based on molar ratio from ICP data.

Sample	K	P	W
$K_{12}[H_2P_2W_{12}O_{48}]\cdot 24H_2O$	13.303	2.150	12.000

In a 250 mL beaker, 200 mL of H_2O was mixed with 6.0 mL of AcOH, followed by 9.0 g (88 mmol) $LiOAc{\cdot}2H_2O$. After 5 minutes of stirring and full dissolution, 4.24 g (100 mmol) LiCl was added and stirred for another 5 minutes until full dissolution. 5.6 g (1.42 mmol) of compound **2** was added and the solution was left to stir for 40 min under vigorous stirring. Lastly, the solution was left to crystallize in a temperature-controlled room (18° C). $\{P_8W_{48}\}$ crystals start forming at the top of the solvent layer after 24 hours. Unknown precipitate starts forming shortly after. After 8 days, the beaker was swirled with a glass rod and the filtered on a Büchner funnel through an S4 porosity glass sinter funnel and washed with 100 mL of ^{i}PrOH. The white powder was collected and dried in a desiccator for 24 hours. Yield: 712 mg, 48 µmol, 40% based on W. ^{31}P NMR (242.9 MHz; 303 K; 1 M LiCl/D_2O; δ, ppm): -6.9. Element % weight calculated for $K_{28}Li_5H_7P_8W_{48}O_{184}{\cdot}92H_2O$: P, 1.67; W, 59.58; K, 7.39; Li, 0.23. Found: P, 1.65; W, 55.16; K, 6.26; Li, 0.43.

Supplementary Table 3: $\{P_8W_{48}\}$ elemental ratio based on molar ratio from ICP data

Sample	K	Li	P	W
$K_{28}Li_5H_7[P_8W_{48}O_{184}]{\cdot}92H_2O$	25.633	9.990	8.514	48.000

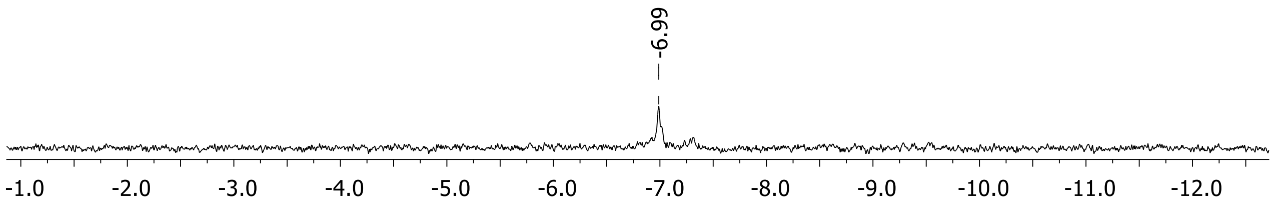

Supplementary Figure 15: ^{31}P NMR of $K_{28}Li_5H_7[P_8W_{48}O_{184}]{\cdot}92H_2O$ synthesised in glassware.

2. Reactionware Cartridge Design and Synthesis of Target Materials

2.1 General Remarks

All reactors used in this study were designed using OpenSCAD[6] (http://www.openscad.org/) based software and printed on Ultimaker 2+ 3D printers[7] (https://ultimaker.com/), with 0.6 mm nozzles using polypropylene from a local supplier. The designs for the synthesis cartridges were exported as stereolithography (.stl) files and translated into 3D printer instruction files using Cura$8 (https://ultimaker.com/en/products/cura-software), a freely available slicer software package developed by Ultimaker. These instruction files were then transferred to the 3D printer for fabrication. Devices were printed at 260°C on 12 mm thick polypropylene plates with 3-layer raft extending 12 mm outside the model footprint to avoid warping. To allow the introduction of necessary reagents, starting materials, or non-printed components the printing process was modified to pause at pre-programmed intervals during the fabrication to allow their placement (see below). Once cartridge fabrication was complete the cartridges were flushed with a suitable inert gas (dry N_2 supplied by BOC) and sealed prior to use.

2.2 Synthesis of (NHS-diazirine) (succinimidyl 4,4'-azipentanoate)

To reduce the risk of any light induced decomposition throughout the synthesis, the modules were covered with aluminium foil whenever practical.

Levulinic acid (0.6 g, 5.17 mmol) dissolved in 2 mL of anhydrous methanol was added into the module 1, through port P_1. The reaction vessel was placed in a cooling bath at -15℃. 7N NH_3 in MeOH (9 mL, 63 mmol, 12 equiv) was then added into the module 1, followed by 3Å molecular sieves (2 g). Port P_1 was then connected to a balloon filled with nitrogen. The reaction was stirred for 3 h. Hydroxylamine-O-sulfonic acid (0.643 g, 5.69 mmol, 1.1 equiv) was dissolved in 4 mL of anhydrous MeOH and added into module 1 dropwise while maintaining the temperature of the reaction at -15℃. The reaction was then stirred for 16 h, while its temperature was allowed to increase to ambient level. The reaction mixture was then transferred to module 2 by applying pressure to port P_1. The solvent was then evaporated by means of vacuum connected to port P_2. The crude product was then re-dissolved in 5 mL of anhydrous MeOH, giving colourless solution. The reaction vessel was placed inside an ice bath and N,N-dimethylethylamine (0.378 g, 5.17 mmol,

0.57 mL, 1 equiv) was introduced, through port P_2.This was stirred for 5 min, after which gradual addition of Iodine beads commenced. When adding the iodine, the colour of the reaction mixture became brown initially, but within a few seconds the colour disappeared to give a colourless solution again. Upon further addition of iodine, the rate of the disappearance of the brown colour slowed significantly. When the brown colour faded within 7 min of the last addition, giving yellow (not brown) tinted solution at the end of the 7 min period, the addition of iodine was stopped. The reaction mixture was stirred for additional 30 min. The ice bath was then removed and 2.4 mL of saturated aqueous solution of potassium iodide was introduced into the module, through port P_2. After further 5 min of stirring, 1.2 mL of saturated aqueous solution of ascorbic acid was introduced. After further 5 min of stirring, gradual addition of 3M HCl (0.85 mL) commenced. Note that the HCl was added in approximately 0.1 mL aliquots. The reaction mixture was then stirred for another 5 min. Slow introduction of 45 mL of diethyl ether was performed through port P_3. Another 8 mL of saturated solution of potassium iodide was also introduced to enhance the recovery of the organic phase from the reaction module. The remaining contents of module 2 were then withdrawn via port P_3 and discarded. Upon addition of the extraction solvent and the potassium iodide solution to module 2, the organic phase containing the product flowed into module 3 containing drying agent ($MgSO_4$). The extract was dried overnight at ambient temperature. The following day, the organic extract was transferred to module 4 by means of air pressure applied to port P_{4aux} of module 3. Once transferred, vacuum was applied to port P_{5aux} in order to evaporate the solvent. When this was completed, the product was dissolved in 8 mL of dry dichloromethane introduced through port P_5. The cartridge monolith was then cooled by means of an ice bath. *N*-(3-dimethylaminopropyl)-*N*′-ethylcarbodiimide hydrochloride (0.455 g, 2.37 mmol) was added to the solution via port P_5, followed by *N*-hydroxysuccinimide (0.273 g, 2.37 mmol). The mixture was stirred for 20 h under the atmosphere of argon. Water (3 mL) was added to the crude reaction mixture and this was stirred for 1 min. The water was then removed using a glass pipette. Note, that any organic phase visible in the pipette was transferred back into the vessel. Washing with water, followed by disposal of the aqueous layer was performed two more times. The organic phase was then transferred to module 5 equipped with a phase separator and filled with 3Å molecular sieves in the compartment below the separator. The dried solution was then transferred into module 6. The solvent was evaporated by means of vacuum connected to port P_{8aux}. The crude product was then re-dissolved in a mixture of 2.5 mL of anhydrous diethyl ether and 0.5 mL of anhydrous MeOH. The cartridge monolith was then cooled at 2°C overnight. The following day liquid was withdrawn from module 6 via port P_8

using a syringe fitted with a small diameter needle. The colourless crystals left behind in module 6 were dried under reduced pressure, taking on an off-white colour. Mass (0.214 g, 0.950 mmol), yield 20%, purity 90%. ^{1}H NMR (600.1 MHz; 303 K; $CDCl_3$; δ, ppm; *J*, Hz): 2.83 (4H; s,), 2.52 (2H; t; 7.8), 1.80 (2H; t; 7.8 Hz) 1.07 (3H, s); ^{13}C{^{1}H} NMR (150.9 MHz, 303 K, $CDCl_3$; δ, ppm): 169.1, 167.7, 29.7, 25.9, 25.7, 24.9, 19.6.

Initially, each step of the reaction was carried in a separate module to ensure that the intermediate reaction products met the expected standards. Optimal cartridge volumes for each step of the entire process were also determined at this stage. Once an optimal architecture and volume was found for each step of the entire synthesis, the modules were combined together one by one stepwise to build up the final sequence. Following this approach an entire monolithic system of cartridges was created and fabricated.

Since the entire synthetic process was enclosed in monolithic system formed out of six cartridges it was necessary to ensure that no undesired transfer of a reaction mixture was possible between neighbouring modules. To ensure this, screw valves were fitted to the siphons located between the vessels. The valves were normally closed, until it became necessary to transfer reaction content from one vessel to another.

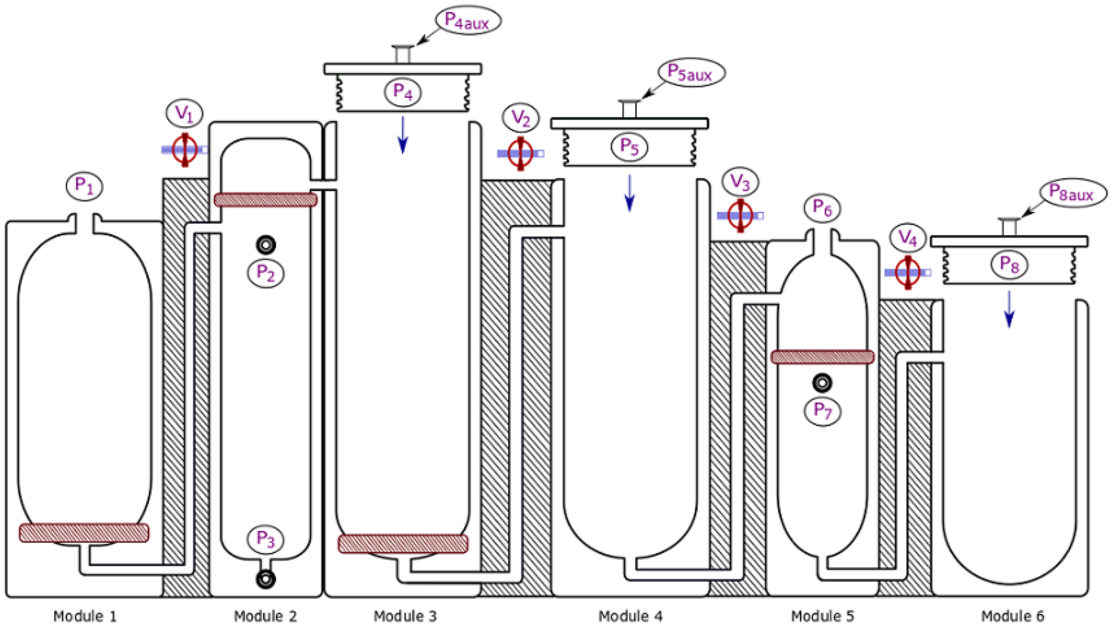

Supplementary Figure 16: Schematic diagram of Cartridge for the synthesis of (NHS-diazirine) (succinimidyl 4,4'-azipentanoate).

Supplementary Table 4: Operation table for the execution of cartridge synthesis of (NHS-diazirine) (succinimidyl 4,4'-azipentanoate).

Time d/hh:mm	Action	P_1	V_1	P_2	P_3	P_4	V_2	P_5	V_3	P_6	P_7	V_4	P_8
0/00:00	Add levulinic acid (0.6 g) dissolved in 2 mL of anhydrous methanol, to module 1, through port P_1	O	C	C	C	C	C	C	C	C	C	C	C
0/00:01	Place the cartridge in a cooling bath at -15℃	O	C	C	C	C	C	C	C	C	C	C	C
0/00:02	Add 9 mL of 7N ammonia in methanol, through port P_1	O	C	C	C	C	C	C	C	C	C	C	C
0/00:03-0/00:05	Add 2 g of 3Å molecular sieves, through port P_1	O	C	C	C	C	C	C	C	C	C	C	C
0/00:06	Connect a nitrogen filled balloon to P_1	O N_2	C	C	C	C	C	C	C	C	C	C	C
0/00:07-0/03:07	Stir for 3 h	O N_2	C	C	C	C	C	C	C	C	C	C	C
0/03:08	Disconnect the balloon from P_1	O	C	C	C	C	C	C	C	C	C	C	C
0/03:09-0/03:19	Add dropwise a solution of hydroxylamine-O-sulfonic acid 0.643 g in 4 mL of dry methanol via a syringe connected to port P_1	O	C	C	C	C	C	C	C	C	C	C	C
0/03:20	Re-connect a nitrogen filled balloon to P_1	O N_2	C	C	C	C	C	C	C	C	C	C	C
0/03:21-0/19:21	Stir for 16 h. Allow gradual increase of the temperature of the bath to ambient level	O N_2	C	C	C	C	C	C	C	C	C	C	C

0/19:22	Disconnect the balloon from P_1	O	C	C	C	C	C	C	C	C	C	C	C
0/19:23-0/19:25	Set valve V_1 to OPEN position	O	O	C	C	C	C	C	C	C	C	C	C
0/19:26	Open port P_2	O	O	O	C	C	C	C	C	C	C	C	C
0/19:27-0/19:37	Apply air pressure to P_1 and transfer the solution from module 1 to module 2	O	O	O	C	C	C	C	C	C	C	C	C
0/19:38	Add 2 mL of dry methanol to module 1, through P_1	O	O	O	C	C	C	C	C	C	C	C	C
0/19:39-0/19:44	Apply air pressure to P_1 and transfer the solution from module 1 to module 2	O	O	O	C	C	C	C	C	C	C	C	C
0/19:45	Close P_1	C	O	O	C	C	C	C	C	C	C	C	C
0/19:46	Set valve V_1 to CLOSED position	C	C	O	C	C	C	C	C	C	C	C	C
0/19:47-1/02:47	Apply vacuum to P_2 and reduce the volume of the liquid in module 2 to aprox 1 mL	C	C	O Vac.	C	C	C	C	C	C	C	C	C
1/02:48	Add 5 mL of dry methanol through P_2	C	C	O	C	C	C	C	C	C	C	C	C
1/02:49-1/02:52	Stir for 3 min	C	C	O	C	C	C	C	C	C	C	C	C
1/02:53	Place the cartridge inside an ice bath at 0°C	C	C	O	C	C	C	C	C	C	C	C	C
1/02:54	Add 0.378 g of N,N-dimethylethylamine through port P_2	C	C	O	C	C	C	C	C	C	C	C	C
1/02:55-1/03:00	Stir for 5 min	C	C	O	C	C	C	C	C	C	C	C	C

1/03:01-<br>1/06:31	Gradually add I_2 as solid through port P_2, while stirring	C	C	O	C	C	C	C	C	C	C	C	C
1/06:32-<br>1/07:02	Stir for additional 30 min after the last addition of I_2	C	C	O	C	C	C	C	C	C	C	C	C
1/07:03	Remove the cartridge from the ice bath	C	C	O	C	C	C	C	C	C	C	C	C
1/07:04-<br>1/07:09	Add 2.4 mL of saturated KI solution, through P_2, and stir for 5 min	C	C	O	C	C	C	C	C	C	C	C	C
1/07:10-<br>1/07:15	Add 1.2 mL of saturated ascorbic acid solution, through P_2, and stir for 5 min	C	C	O	C	C	C	C	C	C	C	C	C
1/07:16-<br>1/07:26	Use port P_2 to add 0.85 mL of 3M HCl in approximately 0.1 mL aliquots while stirring vigorously	C	C	O	C	C	C	C	C	C	C	C	C
1/07:27-<br>1/07:32	Stir for additional 5 min after the addition of the final aliquot of HCl	C	C	O	C	C	C	C	C	C	C	C	C
1/07:33	Close port P_2	C	C	C	C	C	C	C	C	C	C	C	C
1/07:34-<br>1/07:39	Open port P_4 and add 5 g magnesium sulfate to module 3	C	C	C	C	O	C	C	C	C	C	C	C
1/07:40	Close P_4 and open P_4aux	C	C	C	C	O<br>aux	C	C	C	C	C	C	C
1/07:41-<br>1/08:01	Introduce 45 mL of diethyl ether through port P_3 in 9 mL aliquots. Stir for 1 min between each addition.	C	C	C	O	O<br>aux	C	C	C	C	C	C	C

1/08:02	Stop stirring and introduce 8 mL of saturated KI solution through port P_3	C	C	C	O	O aux	C	C	C	C	C	C	C
1/08:03	Withdraw the waste liquid from module 2 through P_3	C	C	C	O	O aux	C	C	C	C	C	C	C
1/08:04	Close ports P_3 and P_4aux	C	C	C	C	C	C	C	C	C	C	C	C
1/08:05-2/00:05	Place the cartridge in a dark place at ambient temperature, to allow for drying of the organic extract.	C	C	C	C	C	C	C	C	C	C	C	C
2/00:06	Open ports P_4aux and P_5aux. Set valve V_2 to OPEN position	C	C	C	C	O aux	O	O aux	C	C	C	C	C
2/00:07-2/00:17	Apply gentle air pressure to P_4aux and transfer the liquid from module 3 to module 4	C	C	C	C	O aux	O	O aux	C	C	C	C	C
2/00:18	Open port P_4 and add 10 mL of dry diethyl ether into module 3	C	C	C	C	O	O	O aux	C	C	C	C	C
2/00:19	Close P_4	C	C	C	C	O aux	O	O aux	C	C	C	C	C
2/00:19-2/00:22	Apply gentle air pressure to port P_4aux and transfer the liquid from module 3 to module 4	C	C	C	C	O aux	O	O aux	C	C	C	C	C
2/00:23	Open port P_4 and add 10 mL of dry diethyl ether into module 3	C	C	C	C	O	O	O aux	C	C	C	C	C
2/00:24	Close port P_4	C	C	C	C	O aux	O	O aux	C	C	C	C	C

2/00:27	Apply gentle air pressure to port P_4aux and transfer the liquid from module 3 to module 4	C	C	C	C	O<br>aux	O	O<br>aux	C	C	C	C	C
2/00:28	Close port P_4aux and valve V_2	C	C	C	C	C	C	O<br>aux	C	C	C	C	C
2/00:29-<br>2/07:29	Apply vacuum to port P_5aux and evaporate the solvent from module 4	C	C	C	C	C	C	O<br>Vac	C	C	C	C	C
2/07:30	Open port P_5 and add 8 mL of anh DCM to module 4	C	C	C	C	C	C	O	C	C	C	C	C
2/07:31-<br>2/07:33	Stir for 1 min then place the cartridge in an ice bath at 0°C	C	C	C	C	C	C	O	C	C	C	C	C
2/07:34	Add EDCI·HCl* (0.455 g) through port P_5 and stir for 1 min	C	C	C	C	C	C	O	C	C	C	C	C
2/07:35	Add NHS** (0.273 g) through port P_5	C	C	C	C	C	C	O	C	C	C	C	C
2/07:36	Close port P_5	C	C	C	C	C	C	C	C	C	C	C	C
2/07:37	Connect nitrogen filled balloon to port P_5aux	C	C	C	C	C	C	O<br>N_2	C	C	C	C	C
2/07:38-<br>3/03:38	Stir for 20 h. Allow the bath temperature to return to ambient level	C	C	C	C	C	C	O<br>N_2	C	C	C	C	C
3/03:39	Open port P_5	C	C	C	C	C	C	O	C	C	C	C	C
3/03:40	Add 3 mL of water to module 4 through port P_5 and stir for 1 min	C	C	C	C	C	C	O	C	C	C	C	C

3/03:41-3/03:43	Remove the water with a Pasteur pipette	C	C	C	C	C	C	O	C	C	C	C	C
3/03:44	Add 3 mL of water to module 4 through port P_5 and stir for 1 min	C	C	C	C	C	C	O	C	C	C	C	C
3/03:45-3/03:47	Remove the water with a Pasteur pipette	C	C	C	C	C	C	O	C	C	C	C	C
3/03:48	Add 3 mL of water to module 4 through port P_5 and stir for 1 min	C	C	C	C	C	C	O	C	C	C	C	C
3/03:49-3/03:51	Remove the water with a Pasteur pipette	C	C	C	C	C	C	O	C	C	C	C	C
3/03:52-3/03:53	Close port P_5 and open port P_5aux	C	C	C	C	C	C	O aux	C	C	C	C	C
3/03:54	Set valve V_3 to OPEN position	C	C	C	C	C	C	O aux	O	C	C	C	C
3/03:55	Open port P_6 and attach a 2 mL syringe without the plunger	C	C	C	C	C	C	O aux	O	O	C	C	C
3/03:56	Open port P_7	C	C	C	C	C	C	O aux	O	O	O	C	C
3/03:57-3/03:59	Attach 5 mL syringe filled with air to port P_5aux and inject the air inside module 4, pushing the liquid to module 5	C	C	C	C	C	C	C	O	O	O	C	C
3/04:00	Attach a syringe to port P_7 and pull the plunger slowly to facilitate transfer of liquid through the phase separator inside module 5	C	C	C	C	C	C	C	O	O	C	C	C

3/04:01	Set valve V_4 to OPEN position	C	C	C	C	C	C	C	O	O	C	O	C
3/04:02	Open port P_8 and close port P_6	C	C	C	C	C	C	C	O	C	C	O	O
3/04:03	Push the plunger of the syringe attached to port P_7 to transfer the liquid from module 5 to module 6	C	C	C	C	C	C	C	O	C	C	O	O
3/04:04	Open port P_6 and fit it with a syringe without the plunger	C	C	C	C	C	C	C	O	O	C	O	O
3/04:05	Use the 5 mL syringe at port P_5aux to inject more air inside module 4, pushing any remaining liquid to module 5	C	C	C	C	C	C	C	O	O	C	O	O
3/04:06	Gently pull the plunger of the syringe attached to port P_7 to facilitate transfer of liquid through the phase separator inside module 5	C	C	C	C	C	C	C	O	O	C	O	O
3/04:07	Close port P_6	C	C	C	C	C	C	C	O	C	C	O	O
3/04:08	Push the plunger of the syringe attached to port P_7 to transfer the liquid from module 5 to module 6	C	C	C	C	C	C	C	O	C	C	O	O
3/04:09	Open port P_5 and add 10 mL of DCM to module 4	C	C	C	C	C	C	O	O	C	C	O	O
3/04:10	Open port P_6 and fit it with a syringe without the plunger	C	C	C	C	C	C	O	O	O	C	O	O

3/04:11	Close port P_5 and use the 5 mL syringe at port P_5aux to inject air inside module 4, pushing the DCM to module 5	C	C	C	C	C	C	C	O	O	C	O	O
3/04:12	Gently pull the plunger of the syringe attached to port P_7 to facilitate transfer of liquid through the phase separator inside module 5	C	C	C	C	C	C	C	O	O	C	O	O
3/04:13	Close port P_6	C	C	C	C	C	C	C	O	C	C	O	O
3/04:14	Push the plunger of the syringe attached to port P_7 to transfer the liquid from module 5 to module 6	C	C	C	C	C	C	C	O	C	C	O	O
3/04:15	Open port P_6 and fit it with a syringe without the plunger	C	C	C	C	C	C	C	O	O	C	O	O
3/04:16	Use the 5 mL syringe at port P_5aux to inject more air inside module 4, pushing any remaining DCM to module 5	C	C	C	C	C	C	C	O	O	C	O	O
3/04:17	Gently pull the plunger of the syringe attached to port P_7 to facilitate transfer of liquid through the phase separator inside module 5	C	C	C	C	C	C	C	O	O	C	O	O
3/04:18	Close port P_6	C	C	C	C	C	C	C	O	C	C	O	O

3/04:19	Push the plunger of the syringe attached to port P_7 to transfer the liquid from module 5 to module 6	C	C	C	C	C	C	C	O	C	C	O	O
3/04:20	Open port P_6 and fit it with a syringe without the plunger	C	C	C	C	C	C	C	O	O	C	O	O
3/04:21	Open port P_5 and add 10 mL of DCM to module 4	C	C	C	C	C	C	O	O	O	C	O	O
3/04:22	Close port P_5 and use the 5 mL syringe at port P_5aux to inject air inside module 4, pushing the DCM to module 5	C	C	C	C	C	C	C	O	O	C	O	O
3/04:24	Gently pull the plunger of the syringe attached to port P_7 to facilitate transfer of liquid through the phase separator inside module 5	C	C	C	C	C	C	C	O	O	C	O	O
3/04:25	Close port P_6	C	C	C	C	C	C	C	O	C	C	O	O
3/04:26	Push the plunger of the syringe attached to port P_7 to transfer the liquid from module 5 to module 6	C	C	C	C	C	C	C	O	C	C	O	O
3/04:27	Open port P_6 and fit it with a syringe without the plunger	C	C	C	C	C	C	C	O	O	C	O	O
3/04:28	Use the 5 mL syringe at port P_5aux to inject more air inside module 4, pushing any remaining DCM to module 5	C	C	C	C	C	C	C	O	O	C	O	O

3/04:29	Gently pull the plunger of the syringe attached to port P_7 to facilitate transfer of liquid through the phase separator inside module 5	C	C	C	C	C	C	C	O	O	C	O	O
3/04:30	Close port P_6	C	C	C	C	C	C	C	O	C	C	O	O
3/04:31	Push the plunger of the syringe attached to port P_7 to transfer the liquid from module 5 to module 6	C	C	C	C	C	C	C	O	C	C	O	O
3/04:32-3/04:36	Close all ports and open port P_8aux	C	C	C	C	C	C	C	C	C	C	C	O aux
3/04:37-3/11:37	Connect vacuum to port P_8aux and evaporate the solvent from module 6	C	C	C	C	C	C	C	C	C	C	C	O aux Vac
3/11:38	Open port P_8	C	C	C	C	C	C	C	C	C	C	C	O
3/11:39	Add 2.5 mL of anh diethyl ether and 0.5 mL of anh methanol into module 6	C	C	C	C	C	C	C	C	C	C	C	O
3/11:40-3/11:44	Stir until dissolved***	C	C	C	C	C	C	C	C	C	C	C	O
3/11:45	Close port P_8 and connect a balloon filled with nitrogen gas to port P_8aux	C	C	C	C	C	C	C	C	C	C	C	O aux N_2

3/11:46-<br>4/11:46	Place the cartridge at 2°C	C	C	C	C	C	C	C	C	C	C	C	O<br>aux<br>N_2
4/11:47-<br>4/11:49	Open port P_8 and use syringe fitted with a small diameter needle to withdraw the mother liquor from module 6. NOTE: Use a needle with a crumpled end	C	C	C	C	C	C	C	C	C	C	C	O
4/11:50-<br>4/12:00	Pre-dry the crystals of the product with a gentle flow of nitrogen gas	C	C	C	C	C	C	C	C	C	C	C	O
4/12:01	Close port P_8 and connect vacuum to port P_8aux	C	C	C	C	C	C	C	C	C	C	C	O<br>aux<br>Vac
4/12:02-<br>4/17:02	Dry the product under vacuum	C	C	C	C	C	C	C	C	C	C	C	O<br>aux<br>Vac
	Store in the dark	C	C	C	C	C	C	C	C	C	C	C	C
Complete													

**N*-(3-dimethylaminopropyl)-*N'*-ethylcarbodiimide hydrochloride
***N*-hydroxysuccinimide
***If complete dissolution does not occur, add another 1.25 mL of and diethyl ether and 0.25 mL of anhydrous methanol

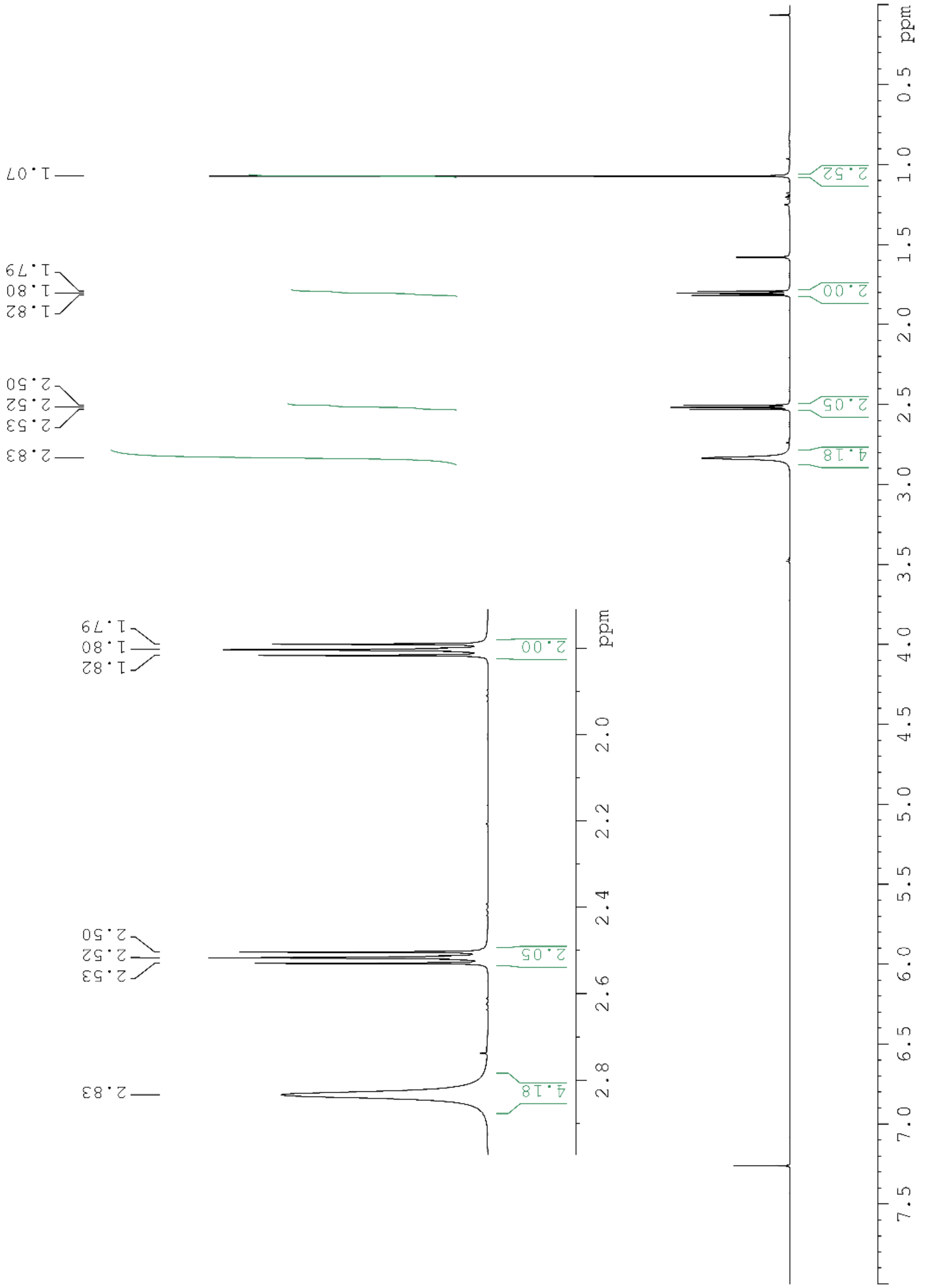

Supplementary Figure 17: ^{1}H NMR of (succinimidyl 4,4′-azipentanoate) synthesised in reactionware cartridge.

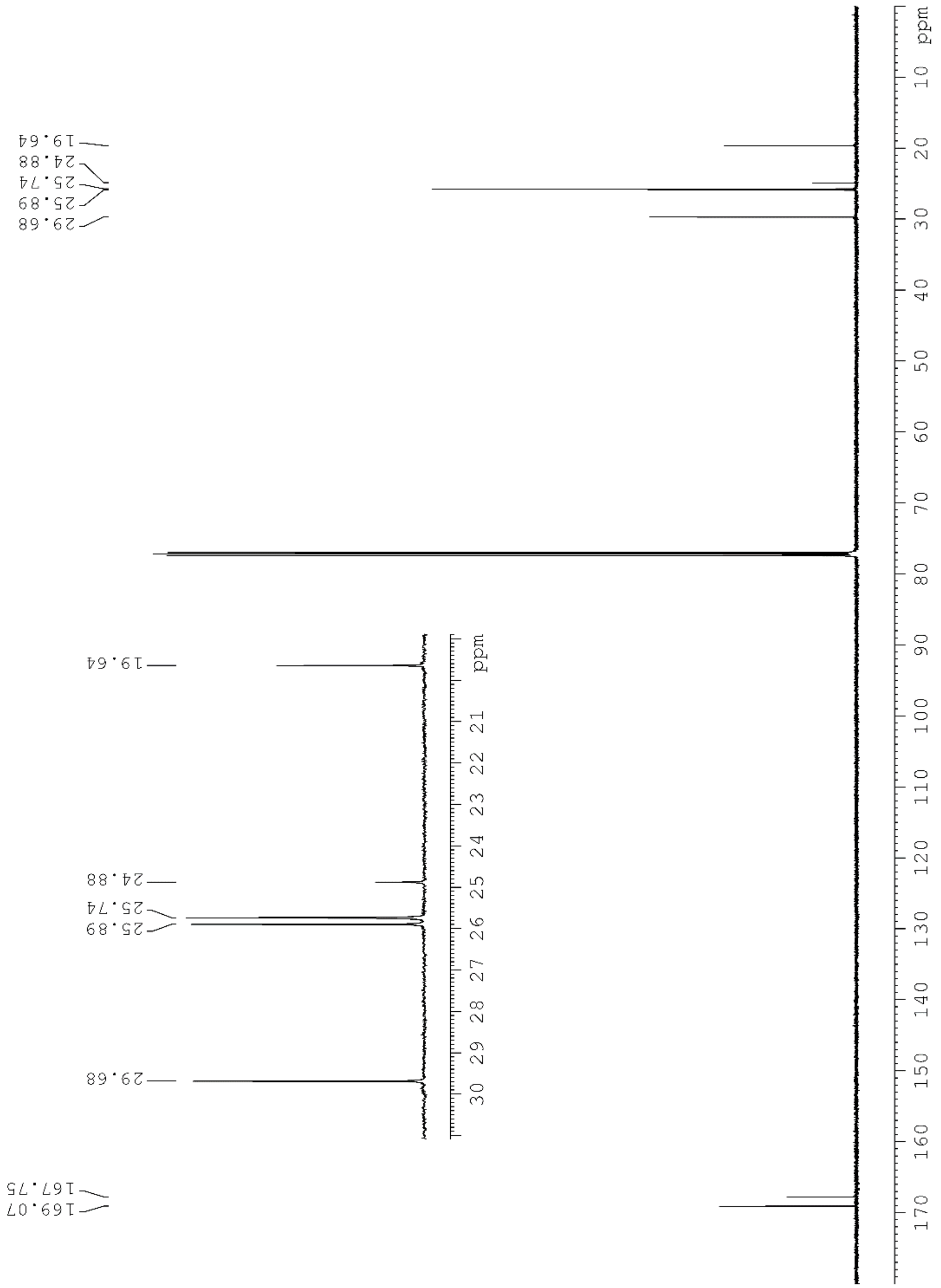

Supplementary Figure 18:$^{13}C\{^{1}H\}$ NMR of (succinimidyl 4,4′-azipentanoate) synthesised in reactionware cartridge.

2.3 Synthesis of tris(dibenzylideneacetone)dipalladium(0)

Step 1. Benzaldehyde (169.7 mg, 1.599 mmol, 2 equiv) was introduced into module 1 via port P2, this was followed by HPLC grade acetone (46.46 mg, 59.3 µL, 0.7999 mmol, 1 equiv) and 1 mL of EtOH. The mixture was stirred, while 10% w/v NaOH (1.44 mL, 3.60 mmol, 4.5 equiv) was added dropwise over the course of 30 min. The reaction was stirred for further 2 h. The waste liquid was then withdrawn via valve V1 leaving behind the yellow precipitate of the product. The crude was washed three times with 6 mL of water, the water withdrawn again through valve V1 and the product dried overnight under vacuum.

Step 2. Palladium acetate (80 mg, 0.356 mmol) was loaded into the module 1 containing dibenzylideneacetone, followed by addition of sodium acetate (292.3 mg, 3.56 mmol) and 8 mL of anhydrous methanol. This mixture was stirred for 3.5 h under argon atmosphere in heat stabilised water bath. The internal temperature of the reaction was maintained at 40℃. During this time dark brown precipitate was formed. The reaction vessel was cooled down to ambient temperature and the precipitate filtered off and washed twice with 6 mL of water, then once with 3 mL of MeOH and once with 3 ml of anhydrous MeOH. The crude was dried thoroughly under vacuum and then re-dissolved in 25 mL of dry chloroform. This solution was transferred to module 2 and the volume of the chloroform was reduced to 5 mL by evaporation. 20 mL of HPLC grade acetone was then added into the solution of the product in chloroform. The resultant mixture was kept at -18℃ overnight. The following day the dark brown solid was filtered off and washed twice with 5 mL of cold acetone. The product was dried thoroughly under vacuum yielding 175 mg (0.169 mmol, 95%) of the target product with 96% purity according to the ^{1}H NMR. ^{1}H NMR (600.1 MHz; 303 K; $CDCl_3$; δ, ppm): Major isomer- 6.80, 6.73, 6.64, 6.45, 6.35, 6.15, 5.93, 5.89, 5.33, 5.13, 4.98, 4.93; Minor isomer– 6.03, 5.97, 5.65, 5.03, 4.91, 4.85;

The approach to cartridge optimisation in the case of Pd_2dba_3, was similar to that taken for the diazirine synthesis. First, each step of the synthetic process was carried in separate cartridges, to ensure that each of them performed their function well, before they were assembled into a single monolith. It is worth mentioning that the heat transfer through polypropylene walls is slower compared to that through glass walls. To ensure that the components of the reaction had enough time to react, the time of the reactions in the case of the polypropylene vessels, was counted from

the moment the reaction mixture reached the target temperature, yielding slight increase of reaction times.

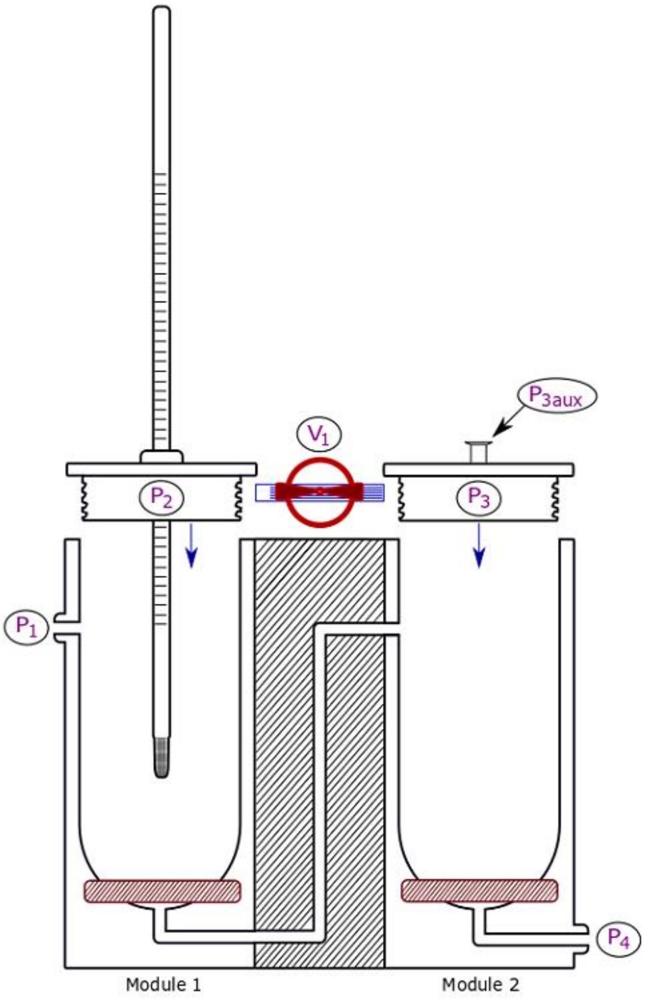

Supplementary Figure 19: Schematic diagram of Cartridge for the synthesis of tris(dibenzylideneacetone)dipalladium(0).

Supplementary Table 5: Operation table for the execution of cartridge synthesis of tris(dibenzylideneacetone)dipalladium(0).

Time d/hh:mm	Action	P_1	P_2	V_1	P_3	P_4
0/00:00	Add EtOH (1 mL) to module 1, through port P_2	C	O	C	C	C
0/00:01	Add benzaldehyde (169.7 mg)) to module 1, through port P_2	C	O	C	C	C

0/00:02	Add acetone (59.3 uL)) to module 1, through port P_2	C	O	C	C	C
0/00:03	Stir	C	O	C	C	C
0/00:04-0/00:34	Add 10% NaOH (aq), dropwise (1.44 mL),) to module 1, through port P_2	C	O	C	C	C
0/00:35-0/02:35	Stir	C	O	C	C	C
0/02:35-0/02:45	Remove valve V_1 completely, use vacuum suction to pull liquid waste from the module 1	C	O	O Vacuum on	C	C
0/02:46	Add deionised water (6 mL) to module 1	C	O	O Vacuum off	C	C
0/02:47-0/02:49	Stir	C	O	O Vacuum off	C	C
0/02:49-0/02:54	Use vacuum suction to pull liquid waste from module 1 via port V_1	C	O	O Vacuum on	C	C
0/02:55	Add deionised water (6 mL) to module 1, through port P_2	C	O	O Vacuum off	C	C
0/02:56-0/02:58	Stir	C	O	O Vacuum off	C	C
0/02:59-0/03:04	Use vacuum suction to pull liquid waste from module 1 via port V_1	C	O	O Vacuum on	C	C

0/03:05	Add deionised water (6 mL) to module 1, through port P_2	C	O	O Vacuum off	C	C
0/03:06-0/03:08	Stir	C	O	O Vacuum off	C	C
0/03:09-0/03:14	Use vacuum suction to pull liquid waste from module 1 via port V_1	C	O	O Vacuum on	C	C
0/03:15-0/19:15	Place the cartridge in a desiccator and dry the product under vacuum	C	O	O	C	C
0/19:15-0/19:20	Replace valve V_1 and set to CLOSED position	C	O	C	C	C
0/19:21	Add palladium acetate (80 mg) to module 1	C	O	C	C	C
0/19:22	Add sodium acetate (292.3 mg) to module 1	C	O	C	C	C
0/19:23	Add anhydrous MeOH (8 mL) to module 1	C	O	C	C	C
0/19:24	Open port P_1	O	O	C	C	C
0/19:25	Close port P_2	O	C	C	C	C
0/19:26-0/19:28	Flush vessel one with argon gas via port P_2	O	C	C	C	C
0/19:29	Connect a weakly inflated balloon with argon to port P_1	O Argon	C	C	C	C
0/19:30	Place the cartridge in a temperature stabilised oil bath set to achieve 40°C in the reaction mixture.	O Argon	C	C	C	C

0/19:31-<br>0/23:01	Stir	O<br>Argon	C	C	C	C
0/23:02-<br>0/23:32	Stop stirring and cool down to ambient temperature	O<br>Argon	C	C	C	C
0/23:33	Disconnect argon balloon from port P_1 and open port P_2	O	O	C	C	C
0/23:33-<br>0/23:43	Remove valve V_1 completely, use vacuum suction to pull liquid waste from module 1	O	O	O<br>Vacuum<br>on	C	C
0/23:44	Add deionised water (6 mL) to module 1	O	O	O<br>Vacuum<br>off	C	C
0/23:45-<br>0/23:50	Use vacuum suction to pull liquid waste from module 1 via port V_1	O	O	O<br>Vacuum<br>on	C	C
0/23:51	Add deionised water (6 mL) to module 1	O	O	O<br>Vacuum<br>off	C	C
0/23:52-<br>0/23:57	Use vacuum suction to pull liquid waste from module 1 via port V_1	O	O	O<br>Vacuum<br>on	C	C
0/23:58	Add MeOH (3 mL) to module 1	O	O	O<br>Vacuum<br>off	C	C

0/23:59-1/00:04	Use vacuum suction to pull liquid waste from module 1 via port V_1	O	O	O Vacuum on	C	C
1/00:05	Add anhydrous (3 mL) MeOH to module 1	O	O	O Vacuum off	C	C
1/00:06-1/00:11	Use vacuum suction to pull liquid waste from module 1 via port V_1	O	O	O Vacuum on	C	C
1/00:11-1/22:11	Place the cartridge in a desiccator and dry the product under vacuum	O	O	O	C	C
1/22:12-1/22:17	Replace valve V_1 and set to CLOSED position	O	O	C	C	C
1/22:18	Close port P_1	C	O	C	C	C
1/22:19-1/22:29	Re-dissolve the product in a dry, HCl free chloroform (15 mL)	C	O	C	C	C
1/22:30	Set valve V_1 to OPEN position	C	O	O	C	C
1/22:31	Open the auxiliary vacuum port P_3aux	C	O	O	O aux	C
1/22:32	Connect vacuum to port P_3aux	C	O	O	O aux	C
1/22:33-1/22:43	Transfer the dissolved product from module 1 to module 2 by applying vacuum to port P_3aux	C	O	O	O aux	C

1/22:44	Add a portion of a dry, HCl free chloroform (10 mL) to module 1, through port P_2	C	O	O	O aux	C
1/22:45	Stir	C	O	O	O aux	C
1/22:46-1/22:56	Transfer the liquid from module 1 to module 2 by applying vacuum to port P_3aux	C	O	O	O aux	C
1/22:57	Add a portion of a dry, HCl free chloroform (10 mL) to module 1, through port P_2	C	O	O	O aux	C
1/22:58	Stir	C	O	O	O aux	C
1/22:59-1/23:09	Transfer the liquid from module 1 to module 2 by applying vacuum to port P_3aux	C	O	O	O aux	C
1/23:10	Close port P_2	C	C	O	O aux	C
2/00:11	Set valve V_1 to CLOSED position	C	C	C	O aux	C
2/00:12-2/05:12	Apply vacuum to port P_3aux to reduce the volume of chloroform in vessel two to approximately 5 mL	C	C	C	O aux	C
2/05:13	Open port P_3	C	C	C	O	C
2/05:14	Add 20 mL of HPLC grade acetone to module 2	C	C	C	O	C
2/05:15	Stir	C	C	C	O	C

2/05:16	Close port P_3	C	C	C	O aux	C
2/05:17	Connect a balloon filled with argon gas to port P_3aux	C	C	C	O Argon	C
2/05:18-3/03:18	Place the cartridge at -18℃	C	C	C	O Argon	C
3/03:19	Open port P_3	C	C	C	O	C
3/03:20	Open port P_4	C	C	C	O	O
3/03:21-3/03:31	Withdraw liquid from module 2 by applying vacuum to port P_4	C	C	C	O	O Vacuum on
3/03:32	Add 5 mL of cold HPLC grade acetone to module 2	C	C	C	O	O Vacuum Off
3/03:33-3/03:35	Withdraw liquid from module 2 by applying vacuum to port P_4	C	C	C	O	O Vacuum On
3/03:36	Add 5 mL of cold HPLC grade acetone to module 2	C	C	C	O	O Vacuum Off
3/03:37-3/03:42	Withdraw liquid from module 2 by applying vacuum to port P_4	C	C	C	O	O Vacuum On
3/03:43	Disconnect vacuum from port P_4 and close the port	C	C	C	O	C
3/03:44	Close port P_3 and open port P_3aux	C	C	C	O aux	C

3/03:45	Place the cartridge in an oil bath set to 40°C	C	C	C	O<br>aux	C
3/03:46-3/11:46	Connect vacuum to port P_3aux to evaporate any solvent traces from the product	C	C	C	O<br>aux	C
	Store at 2°C under argon gas	C	C	C	C	C
Complete						

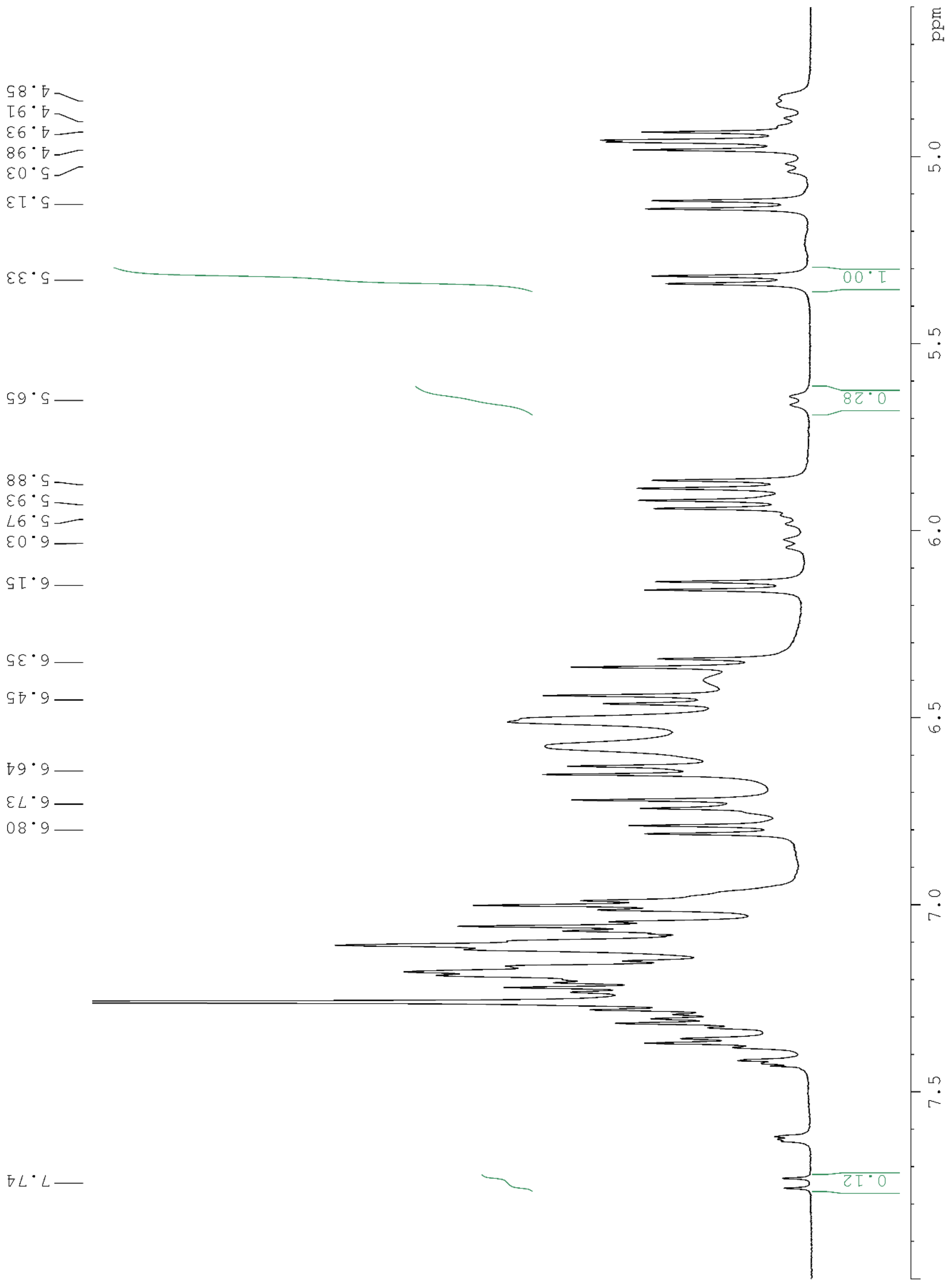

Supplementary Figure 20: ^{1}H NMR of tris(dibenzylideneacetone)dipalladium(0)-chloroform adduct synthesised in reactionware cartridge.

2.4 Synthesis of 1,1,1-triacetoxy-1,1-dihydro-1,2-benziodoxol-3(1H)-one (Dess Martin Periodinane)

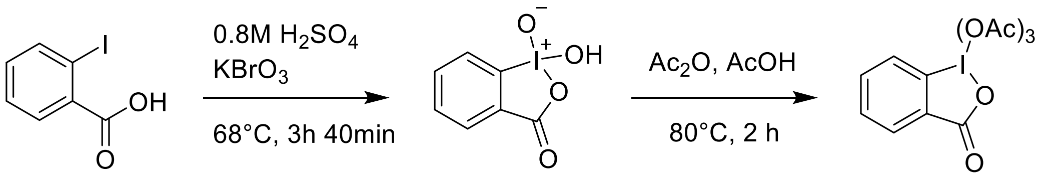

Step 1. 2-iodobenzoic acid (2 g, 8.06 mmol) was loaded into the reaction module fitted with a thermometer, followed by addition of 15 mL of 0.8M sulfuric acid. The resulting suspension was stirred vigorously and brought to 55℃. Potassium bromate was then added in portions (1.785 g, 10.69 mmol, 1.3 equiv) over period of 40 min, followed by additional 11 mL of 0.8M sulfuric acid. The suspension was brought to 68℃ and the reaction stirred for 3 h 40 min. Upon completion, the reaction vessel was cooled to room temperature. The liquid was then withdrawn from the reaction module through port P3, leaving behind the crude IBX in the form of a white powder. Subsequent washing with 30 mL of water, 12 mL of ethanol, followed by 12 mL of anhydrous diethyl ether, afforded the product, which was thoroughly dried under vacuum and used in the following step.

Step 2. Acetic anhydride (4.869 g, 4.5 mL, 47.69 mmol, 5.9 equiv) was added into the reaction module containing dry IBX, this was followed by addition of acetic acid (4.41 g, 4.2 mL, 73.44 mmol, 9.11 equiv). The reaction module was closed with a polypropylene screw cap fitted with a thermometer, to monitor the temperature of the reaction mixture. The vessel was flushed with argon via a port P1. The mixture was heated up to 80℃ and stirred for 2 h. Note that the adjacent chamber was filled with a fused calcium chloride to ensure that no airborne moisture could reach the reaction mixture. When the heating and stirring stopped, the reaction was placed under inert atmosphere and cooled at 2°C, overnight. The following day, liquid was withdrawn from the reaction module, leaving behind white crystals of the product. The crystals were washed with 12 mL of cold anhydrous diethyl ether and dried under vacuum, affording the final product. Mass (2.35 g, 5.541 mmol), yield 69%, purity 81%. ^{1}H NMR (600.1 MHz; 303 K; $CDCl_3$; δ, ppm; *J*, Hz): 8.30 (1H; d; 7.5), 8.28 (1H; d; 8.3), 8.07 (1H; t; 7.8), 7.90 (1H; t; 7.4), 2.32 (3H; s), 2.00 (6H; s); ^{13}C{^{1}H} NMR (150.9 MHz, 303 K, $CDCl_3$; δ, ppm): 175.9, 174.2, 166.3, 142.5, 135.9, 133.9, 132.0, 126.6, 126.2, 20.6, 20.4

The experimental setup for the glassware synthesis required fitting of a drying tube to the reaction flask. In order to include a similar functionality to the reactionware vessel, a side chamber was

added to the upper part of the main chamber. The smaller side chamber was then filled in with a drying agent, such as fused calcium chloride. To stop any drying agent falling into the main reaction chamber, some cotton wool was placed in the channel connecting both chambers.

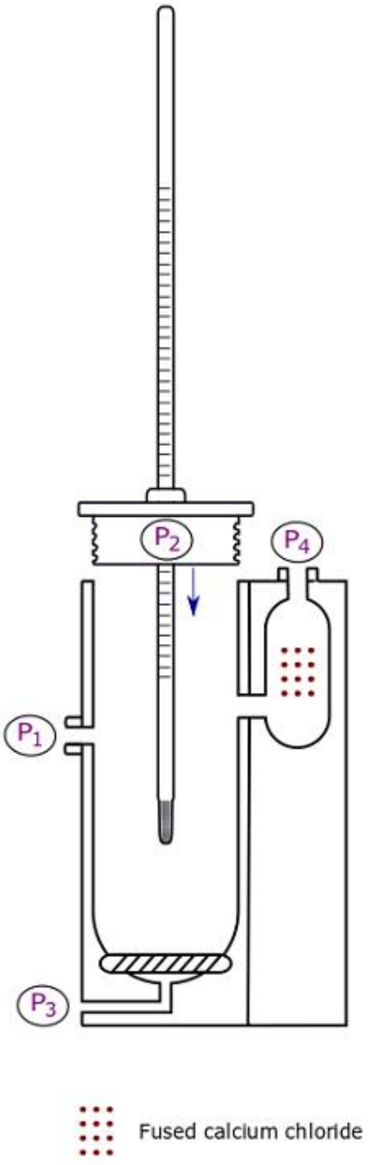

Supplementary Figure 21: Schematic diagram of Cartridge for the synthesis of 1,1,1-triacetoxy-1,1-dihydro-1,2-benziodoxol-3(1H)-one (Dess Martin Periodinane).

Supplementary Table 6: Operation table for the execution of cartridge synthesis of 1,1,1-triacetoxy-1,1-dihydro-1,2-benziodoxol-3(1H)-one (Dess Martin Periodinane).

Time d/hh:mm	Action	P_1	P_2	P_3	P_4

0/00:00	Add 2-iodobenzoic acid through port P_2	C	O	C	O
0/00:01	Add 15 mL of 0.8M sulfuric acid, through port P_2	C	O	C	O
0/00:02	Place the cartridge into an oil bath	C	O	C	O
0/00:03	Close port P_2	C	C	C	O
0/00:04	Set the bath temperature to 60℃	C	C	C	O
0/00:05-0/00:45	Stir at 750 rpm	C	C	C	O
0/00:46	Open port P_1 and port P_2	O	O	C	O
0/00:46-0/01:26	Add potassium bromate (1.785 g) via port P_2 in portions over a period of 40 min. Close port 2 after each addition of potassium bromate. Add additional 11 mL of 0.8M sulfuric acid after addition of potassium bromate is complete.	O	O/C	C	O
0/01:27-0/05:07	Increase the temperature of the bath to achieve 68℃ in the reaction mixture. Stir for 3 h 40 min	O	C	C	O
0/05:08	Stop stirring and remove the cartridge from the oil bath	O	C	C	O
0/05:09-0/06:09	Cool the reaction to room temperature	O	C	C	O
0/06:10	Open ports P_1 and P_3	O	O	O	O
0/06:10-0/06:20	Withdraw the liquid from the module by applying vacuum to port P_3	O	O	O<br>Vacuum<br>On	O

0/06:21	Close port P_3	O	O	O Vacuum Off	O
0/06:22	Add 30 mL of deionised water to the reaction module	O	O	O Vacuum Off	O
0/06:23	Stir	O	O	O Vacuum Off	O
0/06:24-0/06:29	Withdraw the liquid from the module by applying vacuum to port P_3	O	O	O Vacuum On	O
0/06:30	Close port P_3	O	O	O Vacuum Off	O
0/06:31	Add 12 mL of ethanol to the reaction module	O	O	O Vacuum Off	O
0/06:32	Stir	O	O	O Vacuum Off	O
0/06:33-0/06:38	Withdraw the liquid from the module by applying vacuum to port P_3	O	O	O Vacuum On	O
0/06:39	Close port P_3	O	O	O Vacuum Off	O

0/06:40	Add 12 mL of anhydrous diethyl ether to the reaction module	O	O	O<br>Vacuum<br>Off	O
0/06:41-<br>0/06:46	Withdraw the liquid from the module by applying vacuum to port P_3	O	O	O<br>Vacuum<br>On	O
0/06:47-<br>0/23:47	Place the cartridge in a desiccator and dry the product under vacuum	O	O	O	O
0/23:48-<br>0/23:53	Fill the drying module with fused calcium chloride	O	O	O	O
0/23:54	Close port P_3	O	O	C	O
0/23:55	Add acetic anhydride 4.5 mL to the reaction module, through port P_2	O	O	C	O
0/23:56	Add acetic acid 4.2 mL to the reaction module, through port P_2	O	O	C	O
0/23:57	Close port P_2	O	C	C	O
0/23:58-<br>1/00:01	Flush the module with argon gas via port P_1	C	C	C	O
1/00:02	Close port P_1	C	C	C	O
1/00:03	Place the cartridge in an oil bath and start stirring	C	C	C	O
1/00:04	Set the temperature of the bath to achieve 80°C in the reaction mixture.	C	C	C	O
1/00:05-<br>1/02:35	Stop stirring and remove the cartridge from the oil bath	C	C	C	O
1/02:36	Connect an argon filled balloon to port P_1	O<br>Argon	C	C	O

1/02:37	Close port P_4	O<br>Argon	C	C	C
1/02:38-<br>1/03:38	Cool the cartridge to ambient temperature	O<br>Argon	C	C	C
1/03:39-<br>1/23:39	Place the cartridge at 2°C	O<br>Argon	C	C	C
1/23:40	Stop the cooling	O<br>Argon	C	C	C
1/23:41	Disconnect the argon balloon from port P_1	O	C	C	C
1/23:42	Open port P_4	O	C	C	O
1/23:43	Connect nitrogen gas supply to port P_1 and start a gentle flow of nitrogen gas	O	C	C	O
1/23:44	Open port P_2	O	O	C	O
1/23:45	Open port P_3	O	O	O	O
1/23:45-<br>1/23:50	Connect vacuum to port P_3 and withdraw liquid from the module	O	O	O<br>Vacuum<br>On	O
1/23:51	Add anhydrous diethyl ether to the reaction module, through port P_2	O	O	O<br>Vacuum<br>Off	O
1/23:52-<br>1/23:57	Withdraw the liquid from the reaction module by applying vacuum to port P_3	O	O	O<br>Vacuum<br>On	O
1/23:58-<br>2/00:00	Stop the flow of nitrogen gas. Close ports P_2, P_3 and P_4	O	C	C	C

2/00:01-2/03:01	Connect vacuum to port P_1 to dry the product	O<br>Vacuum<br>On	C	C	C
	Store at -18°C under argon gas	C	C	C	C
Complete					

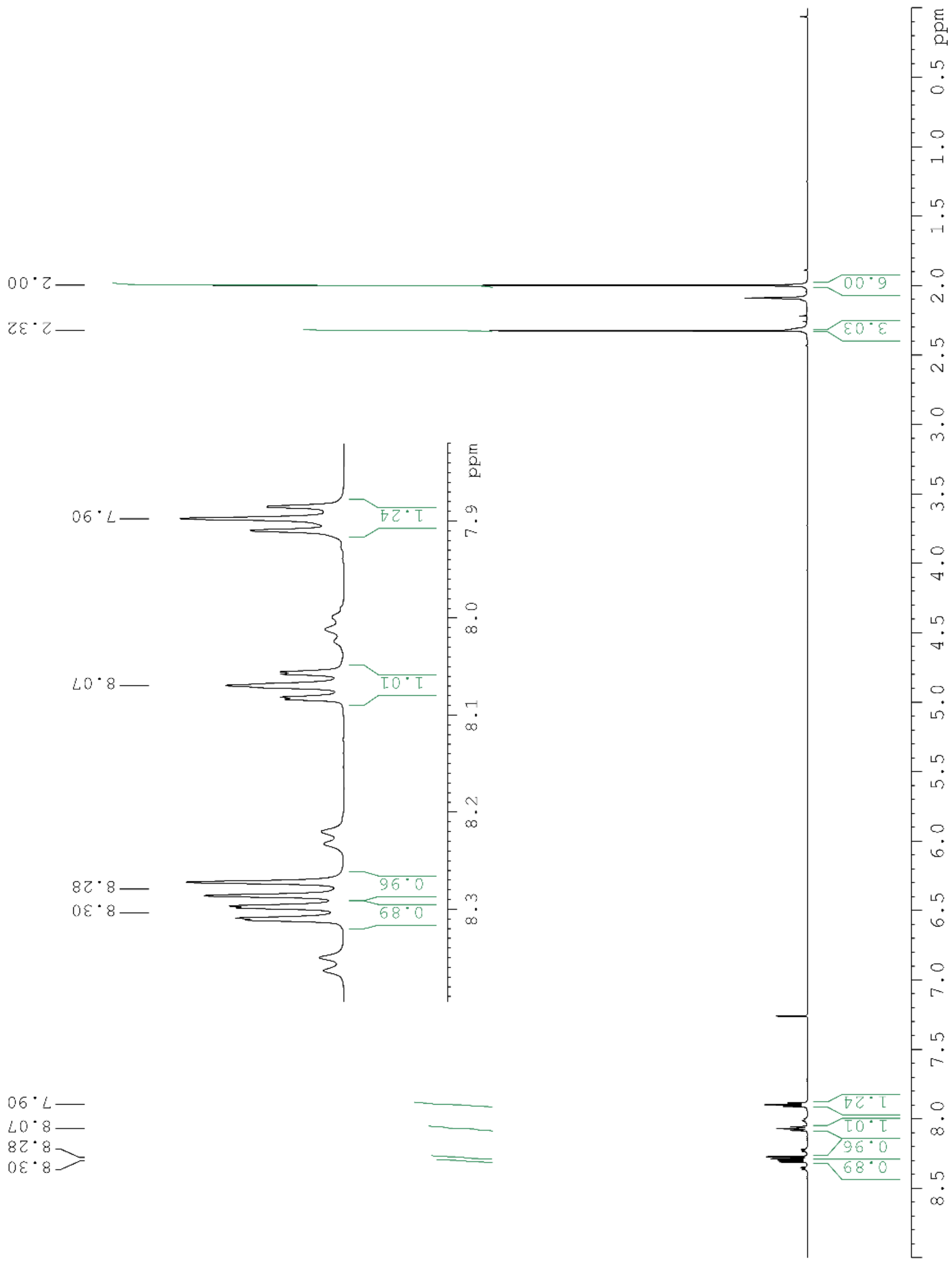

Supplementary Figure 22: ^{1}H NMR of 1,1,1-triacetoxy-1,1-dihydro-1,2-benziodoxol-3(1H)-one synthesised in reactionware cartridge.

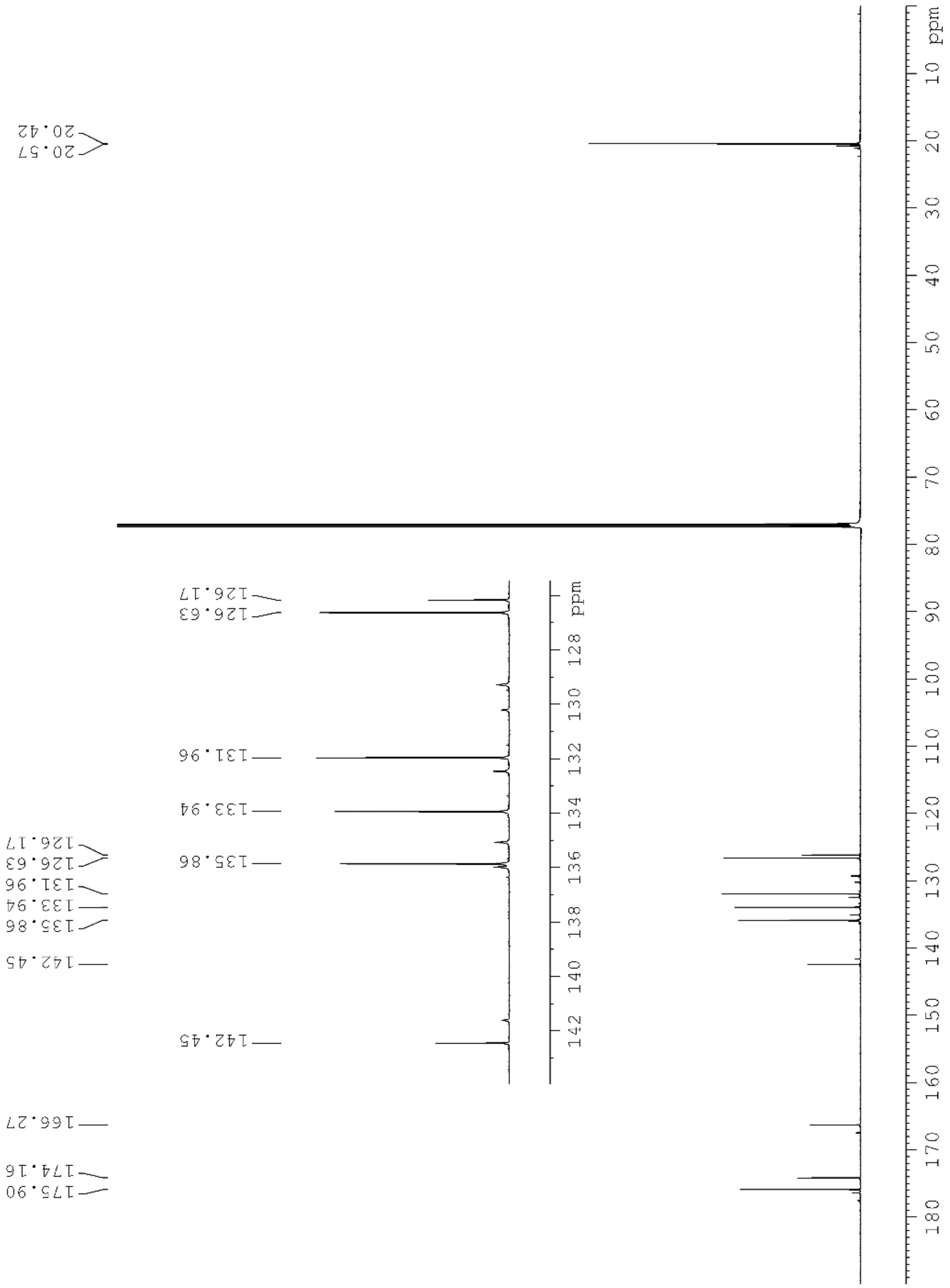

Supplementary Figure 20: $^{13}C\{^{1}H\}$ NMR of 1,1,1-triacetoxy-1,1-dihydro-1,2-benziodoxol-3(1H)-one synthesised in reactionware cartridge.

2.5 Synthesis of $\{P_8W_{48}\}$

Step 1: Both top ports of module (a) are opened to avoid pressure build up. Aqueous Na_2WO_4 (1.7 M, 12.6 g, 43 mmol, pH 5.42) is added to the first reactionware vessel *via* syringe through port 1. A stirrer bar (8 x 1.5 mm) is added. 85% H_3PO_4 (7.6 mL, 146 mmol) is added *via* syringe. Reactionware monolith of modules (a), (b) and (c) is mounted onto a hotplate stirrer plate and the reaction mixture is stirred for 10 minutes until pH stabilises at around 1.9. The PP vessel is then put inside a domestic microwave oven (Daewoo KOR-6A0R, 800 W, 230 V, ~50Hz) preloaded with a beaker of 1 L containing 1L of H_2O. During heating port 2 is left open whilst all other ports are closed to prevent premature liquid transfer to other modules. After heating, the mixture is allowed to cool to room temperature slowly. Port 3 is opened for the addition of NH_4Cl (5.1 g, 95 mmol) and a stirrer bar to module (b). Port 1 is opened and port 2 is closed. Compressed air is flown through port 1 to transfer the reaction mixture from module (a) to module (c). The mixture is stirred for 10 min as a white chalky precipitate forms. Port 5 is opened, port 3 is closed, compressed air is flowed through port 1 to push the mixture from module (b) to module (c) where it is filtered and the filtrate discarded. After filtration port 5 is closed and port 3 is opened. H_2O is added (12.7 mL, 45 °C) to cartridge (b). Port 3 is closed and port 4 is opened and air is flown through port 1 to transfer the lukewarm water from module (b) to (c). The pale green precipitate is dissolved by stirring for 10 minutes. KCl (2 g, 27 mmol) is added to module (c) and the mixture is stirred for 10 min. Port 5 is opened and port 4 closed. Compressed air is applied through port 1 and the reaction mixture is filtered at module (c) and the filtrate discarded. The product is obtained as a pale green powder in module (c). Yield: 4.89 g, 1 mmol, 42.3 % based on W. ^{31}P NMR (242.9 MHz; 303 K; D_2O; δ, ppm): -12.9, -12.3, -11.5.

Step 2: Port 4 was opened. To module (c) lukewarm H_2O (16.7 mL, 45°C) was added through port 4. The precipitate was dissolved under stirring. Port 4 was closed, ports 5, 6 and 7 were opened. Ports 5 and 6 are connected with a tube connector. Compressed air was applied to port 1 and the liquid mixture was transferred from module (c) to module (d). The tube connector is detached. Port 8 was opened and port 6 was closed. A stirrer bar was added through port 8. An aqueous solution of TRIS base (2.7g, 2 M, pH 10.9) was added *via* syringe through port 7. The liquid mixture was stirred at 1000 RPM for 30 min (end pH 8.14). Port 9 was opened ant port 8 was closed. A stirrer bar was added to module (e) through port 9. Compressed air was applied to port 7 to transfer the liquid mixture from module (d) to module (f). KCl (4.5 g, 60 mmol) was added through port 9 to module (e) and the mixture was stirred for 10 min until a colorless solution was obtained. Port 10 was

opened and port 9 was closed. A stirrer bar (25 x 8 mm) was added through port 10. Compressed air was applied to port 7 to transfer the liquid mixture to module (f). An aqueous solution of K_2CO_3 (3.1 g, 2 M, pH 12.5) was added to module (f) through port 10. Solution was stirred vigorously for 20 min (end pH 9.49) and a white chalky precipitate was formed. Port 11 was opened and port 10 was closed. Compressed air was applied through port 7 to filter the solution mixture. The product was afforded as a white powder. Yield: 3.26 g, 0.83 mmol, 83% based on W. ^{31}P NMR (242.9 MHz; 303 K; D_2O; δ, ppm): 2.6, -9.3.

<u>Step 3:</u> Port 10 was opened. To module (f) {P_8W_{48}} buffer (procedure for preparation described below) was added through port 10. The reaction mixture is stirred for 1 hour until full dissolution (pH 4.9). Port 11 was opened and port 10 closed. Compressed air was applied to port 7 and the filtrate was collected in a 150 mL glass beaker. The glass beaker was covered with parafilm and stored in a temperature controlled room (18 °C) to crystallise for a total of 7 days. {P_8W_{48}} crystals start forming as white amorphous blocks. After the crystallisation period the solution mixture is stirred with a glass rod and the contents are filtered on a Buchner funnel with Whatman (grade 1) filter papers. The precipitate is washed with 50 mL of ^{i}PrOH. Product is afforded as a white powder. Recrystallisation for XRD analysis: 660 mg of afforded product was dissolved in 19.5 mL of {P_8W_{48}} buffer. Colorless block crystals formed after 24h. Yield: 895 mg, 60 µmol, 29% based on W. ^{31}P NMR (242.9 MHz; 303 K; 1M LiCl/D_2O; δ, ppm): -6.9. Element % weight calculated for $K_{28}Li_5H_7P_8W_{48}O_{184}\cdot 92H_2O$: P, 1.67; W, 59.58; K, 7.39; Li, 0.23. Found: P, 1.77; W, 63.05; K, 4.92; Li, 0.80.

Supplementary Table 7: {P_8W_{48}} elemental ratio based on molar ratio from ICP data.

Sample	K	Li	P	W
$K_{28}Li_5H_7[P_8W_{48}O_{184}]\cdot 92H_2O$	17.60	16.15	8.00	48.000

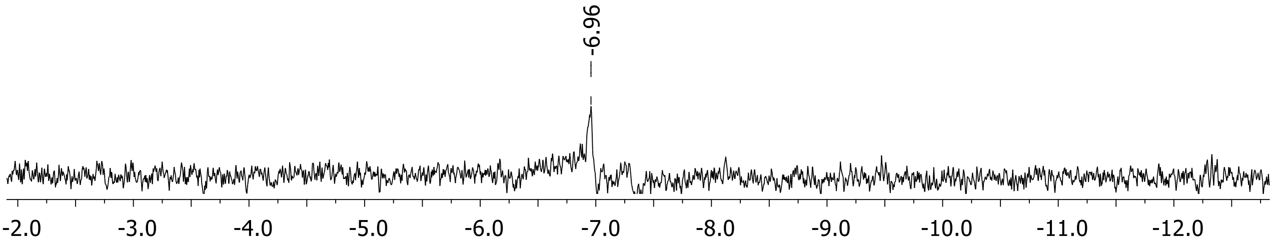

Supplementary Figure 21: ^{31}P NMR of $K_{28}Li_5H_7[P_8W_{48}O_{184}]\cdot 92H_2O$ synthesised in glassware.

Supplementary Table 5. Crystal data and structure refinement for {P_8W_{48}} polyoxometalate.

Identification code	{P_8W_{48}}
Empirical formula	$H_{126}K_{18}Li_{16}O_{244}P_8W_{48}$
Formula	$Li_{16}K_{18}H_6[P_8W_{48}O_{184}](H_2O)_{60}$
Formula weight	13918.40
Temperature	150(2) K
Wavelength	0.71073 Å
Crystal system	Tetragonal
Space group	$I\ 4_{22}$
Unit cell dimensions	a = 27.297(3) Å
	α= 90°.
	b = 27.297(3) Å
	β= 90°.
	c = 20.974(3) Å
	γ= 90°.
Volume	15629(4) Å^3
Z	2
Density (calculated)	2.958 Mg/m^3
Absorption coefficient	17.954 mm^{-1}
F(000)	12280
Crystal size	0.064 x 0.054 x 0.054 mm^3
Theta range for data collection	1.942 to 25.999°.
Index ranges	-17<=h<=33, -33<=k<=30, -25<=l<=25
Reflections collected	58431
Independent reflections	7703 [R(int) = 0.0398]
Completeness to θ = 25.242°	99.9 %
Absorption correction	Analytical
Max. and min. transmission	0.5592 and 0.4758
Refinement method	Full-matrix least-squares on F^2

Data / restraints / parameters	7703 / 0 / 389
Goodness-of-fit on F^2	1.089
Final R indices [$I>2\sigma(I)$]	$R_1 = 0.0233$, w$R_2 = 0.0618$
R indices (all data)	$R_1 = 0.0264$, w$R_2 = 0.0638$
Absolute structure parameter	0.50(2)
Extinction coefficient	0.0000064(17)
Largest diff. peak and hole	1.18 and -0.73 e.Å^{-3}

{P_8W_{48}} buffer preparation: 110.6 mL of H_2O was added (resting pH 5.45) to a 150 mL glass beaker. 6.66 mL of CH_3COOH was added and stirred for 5 minutes. LiOH (2.44 g, 102 mmol) was added to the stirring solution and stirred for a further 5 minutes. LiCl (2.44 g, 58 mmol) was added to the solution under stirring and stirred for another 5 minutes.

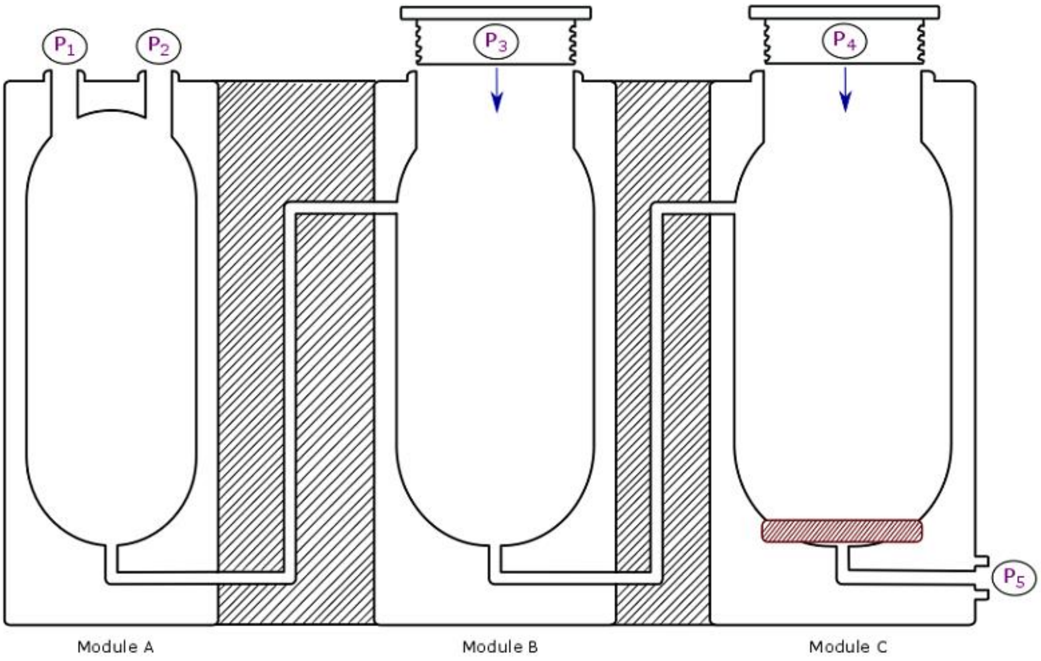

Supplementary Figure 22: Reactionware polypropylene monolith (modules a-c) for the formation of {P_2W_{18}}.

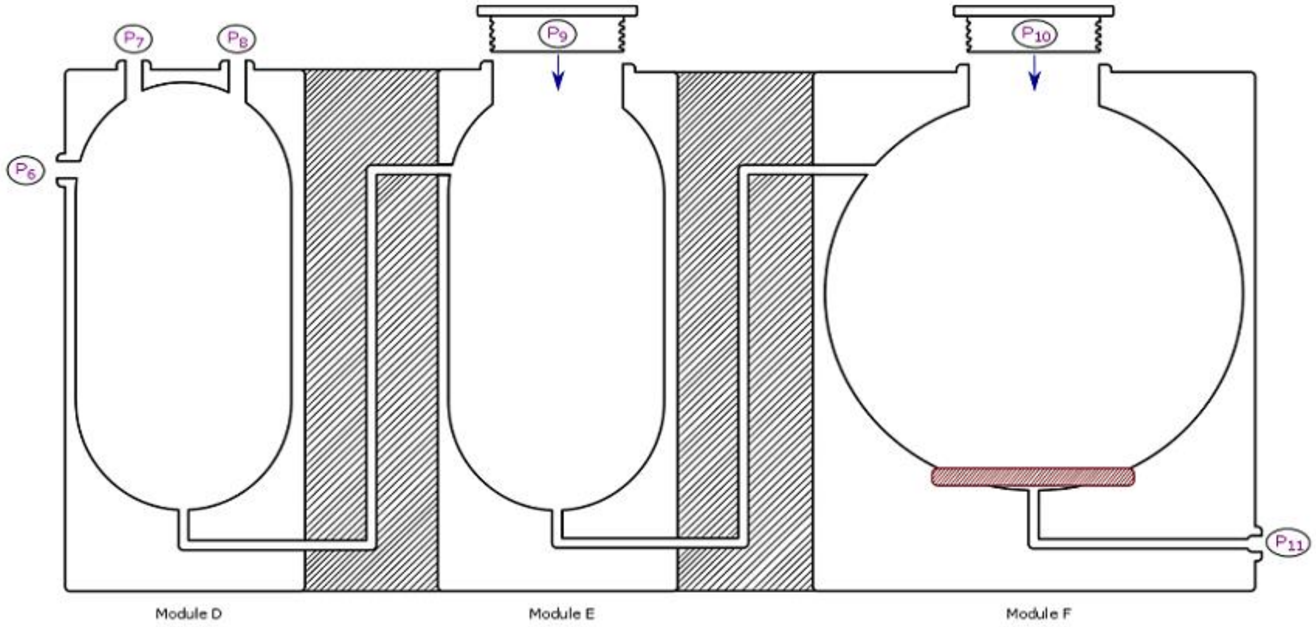

Supplementary Figure 23: Reactionware polypropylene monolith (modules d-f) for the formation of {P_2W_{12}} and {P_8W_{48}}.

Table 8: Operation table for the execution of cartridge synthesis of {P_8W_{48}}.

STEP 1						
Time d/hh:mm	Action	Port				
Prepare prior to synthesis	R1: 12.6 g Na_2WO_4 in 25.3 mL H_2O in syringes	1	2	3	4	5
Prepare prior to synthesis	R2: 7.6 mL 85% H_3PO_4 in a 24 mL syringe	C	C	C	C	C
0/00:00	Open ports 1, 2	O	O	C	C	C
0/00:01	Add stirrer bar through port 1	O	O	C	C	C
0/00:02	Add R1 through port 1	O	O	C	C	C
0/00:04	Add R2 through port 1	O	O	C	C	C
0/00:04 - 0/00:14	Initiate stirring at 1000 RPM for 10 min	O	O	C	C	C
0/00:14	Stop stirring	O	O	C	C	C
0/00:14	Add 1L H_2O beaker to microwave	O	O	C	C	C
0/00:16-0/01:16	Microwave irradiation	C	O	C	C	C
0/01:16-0/01:46	Cool to RT	C	O	C	C	C
0/01:47	Open port 3	C	O	O	C	C
0/01:47	Add stirrer bar to cartridge (b)	C	O	O	C	C
0/01:50	Add 5.1 g of NH_4Cl to cartridge (b)	C	O	O	C	C
0/01:51	Open port 1, close port 2	O	C	O	C	C
0/01:51 - 0/01:53	Apply compressed air to port 1 for liquid mixture transfer	O	C	O	C	C

0/01:53 - 0/02:03	Stir at 1000 RPM for 10 min	O	C	O	C	C
0/02:04	Open port 5, close port 3	O	C	C	C	O
0/02:04 - 0/02:07	Apply compressed air to port 1 for liquid mixture transfer and filtration	O	C	C	C	O
0/02:08	Open port 3, close port 5	O	C	O	C	C
0/02:08	Add H_2O (12.7 mL, 45 °C)	O	C	O	C	C
0/02:11	Open port 4, close port 3	O	C	C	O	C
0/02:12 - 0/02:15	Apply compressed air to port 1 for liquid mixture transfer	O	C	C	O	C
0/02:16	Add stirrer bar to cartridge (c)	O	C	C	O	C
0/02:16 - 0/02:26	Stir at 1000 RPM for 10 min until dissolution	O	C	C	O	C
0/02:26	Add KCl (2 g) through port 4	O	C	C	O	C
0/02:29	Open port 5, close port 4	O	C	C	C	O
0/02:30 - 0/02:33	Apply compressed air to port 1 for liquid mixture filtration	O	C	C	C	O
0/02:34	Close port 5	O	C	C	C	C

**STEP 2**									
Time d/hh:mm	Action	Ports							
		4	5	6	7	8	9	10	11
Prepare prior to synthesis	R3: 2.7 g TRIS base in 11.1 mL H_2O in syringes	C	C	C	C	C	C	C	C
Prepare prior to synthesis	R4: 3.1 g K_2CO_3 in 11.1 mL H_2O in syringes	C	C	C	C	C	C	C	C
0/03:00	Open port 4	O	C	C	C	C	C	C	C
0/03:00	Add H_2O (16.7 mL, 45 °C) to cartridge (c)	O	C	C	C	C	C	C	C

0/03:04	Stir until dissolution at 1000 RPM	O	C	C	C	C	C	C	C
0/03:19	Open ports 5, 6 and 7, close port 4	C	O	O	O	C	C	C	C
0/03:20	Connect ports 5 and 6 with a tube connector	C	O	O	O	C	C	C	C
0/03:20	Apply compressed air to port 1 for liquid transfer	C	O	O	O	C	C	C	C
0/03:23	Detach tube connector	C	O	O	O	C	C	C	C
0/03:24	Open port 8, close ports 4, 5 and 6	C	C	C	O	O	C	C	C
0/03:25	Add stirrer bar to port 8	C	C	C	O	O	C	C	C
0/03:25	Add R3 through port 7	C	C	C	O	O	C	C	C
0/03:27	Stir for 30 min at 1000 RPM	C	C	C	O	O	C	C	C
0/03:57	Open port 9, close port 8	C	C	C	O	C	O	C	C
0/03:57	Add stirrer bar to port 9	C	C	C	O	C	O	C	C
0/03:57	Apply compressed air to port 7 for liquid transfer	C	C	C	O	C	O	C	C
0/04:00	Add KCl (4.5 g) through port 9	C	C	C	O	C	O	C	C
0/04:03	Stir for 10 min until full dissolution	C	C	C	O	C	O	C	C
0/04:13	Open port 10, close port 9	C	C	C	O	C	C	O	C

0/04:16	Add stirrer bar (25 x 8 mm) through port 10	C	C	C	O	C	C	O	C
0/04:16	Apply compressed air for liquid transfer	C	C	C	O	C	C	O	C
0/04:19	Add R4 to port 10	C	C	C	O	C	C	O	C
0/04:21	Stir vigorously for 20 min	C	C	C	O	C	C	O	C
0/04:41	Open port 11, close port 10	C	C	C	O	C	C	C	O
0/04:41	Apply compressed air to port 7 and filter the solution mixture	C	C	C	O	C	C	C	O
0/04:51	Close port 11	C	C	C	O	C	C	C	C
**STEP 3**									
Time d/hh:mm	Action	Ports							
Prepare prior to synthesis	Prepare R5: {P_8W_{48}} buffer	6	7	8	9	10	11		
	Open port 10	C	O	C	C	O	C		
1/00:00	Add R5 through port 10	C	O	C	C	O	C		
1/00:02 - 1/01:02	Stir vigorously for 1 hour	C	O	C	C	O	C		
1/01:02	Open port 11, close port 10	C	O	C	C	C	O		
1/01:03 - 1/01:13	Apply compressed air through port 7	C	O	C	C	C	O		
1/01:13w	Collect filtrate in glass beaker	C	O	C	C	C	O		

3. Product Validation Experiments

3.1 DMP validation. Oxidation of menthol to menthone.[9]

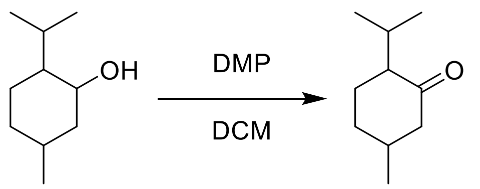

Menthol (0.276 g, 1.77 mmol, 1.5 equiv) was loaded into a round bottom flask and followed by 6 mL of DCM. The content of the flask was stirred to achieve complete dissolution of menthol.

DMP (0.5 g, 1.18 mmol, 1 equiv) was then added in and the vessel was closed with a rubber septum. The reaction mixture was then stirred for 45 min at room temperature. Sodium sulfite 25% w/v (2 mL) was then added to the reaction vessel, along with 2 mL of a saturated aqueous solution of sodium bicarbonate. The resulting mixture was stirred for 30 min. The reaction mixture was extracted with diethyl ether (25 mL), the organic phase dried over magnesium sulfate, followed by filtration and solvent evaporation. Proton NMR of the crude reaction mixture indicated 77% conversion of menthol to menthone in the experiment carried out with DMP synthetized in reactionware and 82% conversion when carried out with the DMP purchased from an external supplier.

3.2 Pd_2dba_3 validation. Suzuki coupling.

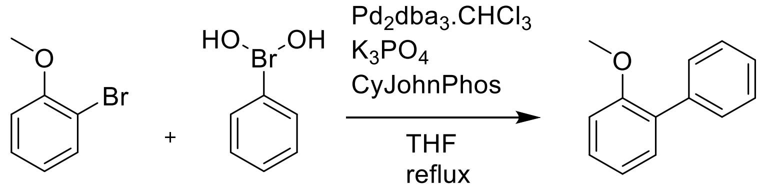

The above Suzuki coupling reaction was carried out according to the published procedure.[10] The experiment carried out with Pd_2dba_3 from an external supplier and experiment carried out with reactionware synthetized catalyst both gave exactly the same conversion ratio to the Suzuki product (94%) by ^{1}H NMR.

3.3 NHS-Diazirine reactivity validation.

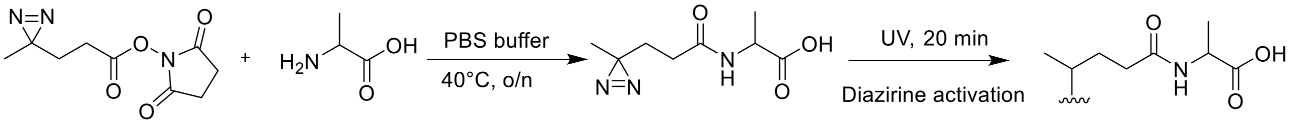

NHS-diazirine (6 mg, 0.027 mmol) and alanine (9.5 mg, 0.1 mmol, 4 equiv) were loaded into a glass vial followed by addition of 0.9 mL of PBS buffer made up in D_2O. The vial was capped and the mixture was stirred at 40 °C overnight. The reaction mixture was then allowed to cool to room temperature, transferred to a quartz vial and stirred while being exposed to UV light (250W Oriel instruments Xe-bulb) for 20 min. A sample of the mixture was analysed by ^{1}H NMR before and after irradiation. These experiments revealed formation of the intermediate) indicated by a shift of the 1.52 ppm doublet peak belonging to free alanine to 1.42 ppm doublet peak belonging to the diazirine alanine intermediate (this diazirine-alanine intermediate was also observed by ESI-MS(+ve) m/z: calc. for $C_8H_{13}N_3O_3$ 200.21 $[M+H]^+$, found 200.152 $[M+H]^+$). The ^{1}H NMR spectrum of the mixture after UV irradiation showed formation of complex mixture of products, though one crucial observation revealed shift of 2.15 ppm triplet of the proton adjacent to the former diazirine moiety to a new position at 2.41 ppm indicating the activation and subsequent reaction of the diazirine moiety.

No differences were observed between experiments conducted with commercially available NHS-diazirine and that synthesised in the reactionware system.

3.4 $\{P_8W_{48}\}$ validation – formation of a wheel-shaped Cu_{20} tungstophosphate

As the $\{P_8W_{48}\}$ moiety is not commercially available, validation was performed by repeating a literature synthesis which uses it as a starting material.[11] The synthesis was carried out as described apart from the filtration procedure. The filtration was performed on a hot solution instead of waiting for it to cool down as suggested in the reference. Once the mother liqueur was prepared, after few hours crystals start to appear. The solution was left to crystallise for a day and blue block crystals of

sufficient quality were collected and analysed using an X-ray diffractometer.Unit cell: a = b = 24.116, c = 21.810; α = β = γ = 90°. Space group I4/M.

The unit cell reported in the literature[8] has the axis of a = b = 26.753, c = 21.810 with α = β = γ = 90°, and the same space group as the unit cell we report – I4/M. There is a small difference in the *a* and *b* axes but there is no ambiguity regarding the incorporation of a Cu cluster within the {P_8W_{48}} framework as shown by X-ray diffraction structure.

Supplementary Table 9. {P_8W_{48}}-Cu elemental ratio based on molar ratio from ICP data.

Sample	Cu	K	Li	P	W
Cu-{P_8W_{48}}	12.77	21.53	7.67	8.20	48.00

Supplementary References

1 Sun, R. *et al.* Simple Light-Triggered Fluorescent Labeling of Silica Nanoparticles for Cellular Imaging Applications. *Chem. – Eur. J.* **23**, 13893-13896, (2017).

2 Liu, X., Xu, X., Pan, L., Zhang, Q. & Liu, Q. Efficient synthesis of trifluoromethylated cyclopentadienes/fulvenes/norbornenes from divinyl ketones. *Org. Biomol. Chem.* **11**, 6703-6706, (2013).

3 Zalesskiy, S. S. & Ananikov, V. P. $Pd_2(dba)_3$ as a Precursor of Soluble Metal Complexes and Nanoparticles: Determination of Palladium Active Species for Catalysis and Synthesis. *Organometallics* **31**, 2302-2309, (2012).

4 Dess, D. B. & Martin, J. C. Readily accessible 12-I-5 oxidant for the conversion of primary and secondary alcohols to aldehydes and ketones. *J. Org. Chem.* **48**, 4155-4156, (1983).

5 Klemperer, W. G. EARLY TRANSITION-METAL POLYOXOANIONS. *Inorg. Synth.* **27**, 71-135, (1990).

6 *OpenSCAD – The Programmers Solid 3D CAD Modeller* < http://www.openscad.org/ > (2019)

7 *Professional 3D printing made accessible |Ultimaker* < https://ultimaker.com/ > (2019)

8 *Ultimaker Cura: Powerful, easy-to-use 3D printing software* < https://ultimaker.com/en/products/cura-software > (2019)

9 Reed, N. A., Rapp, R. D., Hamann, C. S. & Artz, P. G. Circular Dichroism Investigation of Dess–Martin Periodinane Oxidation in the Organic Chemistry Laboratory. *J. Chem. Ed.* **82**, 1053, (2005).

10 Wolfe, J. P., Singer, R. A., Yang, B. H. & Buchwald, S. L. Highly Active Palladium Catalysts for Suzuki Coupling Reactions. *J. Am. Chem. Soc.* **121**, 9550-9561, (1999).

11 Mal, S. S. & Kortz, U. The Wheel-Shaped Cu_{20} Tungstophosphate $[Cu_{20}Cl(OH)_{24}(H_2O)_{12}(P_8W_{48}O_{184})]^{25-}$ Ion. *Angew. Chem. – Int. Ed.* **44**, 3777-3780, (2005).
