# Supplementary material for: 3D designed and printed chemical generators for on demand reagent synthesis
Source: Nat Commun. 2019 Dec 2;10:5496. doi: 10.1038/s41467-019-13328-6 (PMC6889270; doi:10.1038/s41467-019-13328-6)
Supplement: Supplementary file 3 — Description of Additional Supplementary Files [file 41467_2019_13328_MOESM3_ESM.pdf]

Description of Additional Supplementary Files

File Name: Supplementary Data 1

Description:

DMP_catridge.stl -3D printer file for the DMP reactionware

NHS-diazirine_catridge.stl -3D printer file for the NHS-diazirine reactionware

Pd2cba3_catridge.stl -3D printer file for the Pd2cba3 reactionware

P8W48_catridge_part1.stl -3D printer file for the P8W48 reactionware part 1

P8W48_catridge_part1.stl -3D printer file for the P8W48 reactionware part 2
